# A Mathematical Framework for Quantum Hamiltonian Simulation and Duality

**DOI:** 10.1007/s00023-024-01432-3

**Published:** 2024-04-10

**Authors:** Harriet Apel, Toby Cubitt

**Affiliations:** Department of Computer Science, University College London, London, UK

## Abstract

Analogue Hamiltonian simulation is a promising near-term application of quantum computing and has recently been put on a theoretical footing alongside experiencing wide-ranging experimental success. These ideas are closely related to the notion of duality in physics, whereby two superficially different theories are mathematically equivalent in some precise sense. However, existing characterisations of Hamiltonian simulations are not sufficiently general to extend to all dualities in physics. We give a generalised duality definition encompassing dualities transforming a strongly interacting system into a weak one and vice versa. We characterise the dual map on operators and states and prove equivalence ofduality formulated in terms of observables, partition functions and entropies. A building block is a strengthening of earlier results on entropy preserving maps—extensions of Wigner’s celebrated theorem- –to maps that are entropy preserving up to an additive constant. We show such maps decompose as a direct sum of unitary and antiunitary components conjugated by a further unitary, a result that may be of independent mathematical interest.

## Introduction

Duality is a deep straining running throughout physics. Any two systems that are related can be described as being “dual”, up to the strictest sense of duality where all information about one system is recoverable in the other. Calculations or predictions in one theory may be simplified by first mapping to the dual theory, given there is a rigorous relationship between the points of interest. Strong–weak dualities are a common example of this, allowing well-understood perturbation techniques to be leveraged in high energy regimes by considering the dual weak theory [1–4].

In the near-term, there is hope of using quantum computers as analogue simulators to study certain physical properties of quantum many-body systems. In analogue simulation, the Hamiltonian of interest, *H*(*t*), is engineered with a physical system that is then allowed to time evolve continuously. This is in contrast to digital simulation where the time evolution is mapped to quantum circuits— for example, via Trotterisation—which likely requires a scalable, fault tolerant quantum computer [5]. It is believed that analogue simulators without error correction could be sufficient to study interesting physics and this has seen varying experimental success with trapped ions [6], cold atoms in optical lattices [7], liquid and solid state NMR [8], superconducting circuits [9], etc. These artificial systems allow for improved control and simplified measurements compared to in situ materials, providing a promising use for noisy intermediate scale devices.

What it means for one system to ‘simulate’ or ‘be dual to’ another is an important theoretical question, which has only recently begun to be explored. [10] and later [11] gave formal definitions of simulation. Cubitt et al. used this framework to demonstrate certain ‘universal’ spin–lattice models that are able to simulate any quantum many-body system by tuning the interaction parameters. These works consider the strongest possible definition of a duality: all relevant physics is manifestly preserved in the simulator system including measurement outcomes, the partition function and time evolution. While this strengthens [11]’s main result it rules out potentially interesting scenarios where the relationship between the systems’ properties is more subtle.

Of particular interest to physicists are dualities that relate a strongly interacting theory to a weakly interacting one—so-called strong–weak dualities. These dualities are of particular interest as strongly interacting theories beyond the reach of perturbation theory are often challenging to analyse, and strongly interacting phenomena are difficult to elucidate. Strong–weak dualities serve as a valuable tool for addressing these challenges, transforming a strongly interacting system to a dual, weakly interacting system, which is then amendable to perturbation theory. One notable example from particle physics is the phenomenon of S-duality, which relates electric and magnetic descriptions within specific gauge theories. Effectively capturing this and other important classes of dualities in physics, necessitates a more general set of mappings than those previously explored for simulation purposes.

The aim of this work is to explore and extend upon a theoretical framework of duality to better unify operationally how dual systems are related. We significantly generalise the conditions placed on a duality map between operators to allow for operationally valid transformations which crucially include strong–weak dualities. This direction of relaxation is inspired by considering examples of duality studied in physics including the Kramer–Wannier duality [1] and boson–fermion dualities. We derive a full characterisation of these maps and additionally characterise the map on states—which were not shown in previous studies.

Having imposed spectral preserving as a property of dual maps, it follows that other physical properties such as partition functions and entropies are necessarily preserved. A key outcome of this work is the reverse implication: that demanding partition functions (or entropies) are preserved along with convexity is strong enough to predetermine the spectra of the dual maps. This leads to three different definitions of duality that, while seemingly distinct with different domains of application, are in fact mathematically equivalent and are therefore characterised by the same mathematical structure, which we demonstrate. Thus, a duality relationship on any one of these physical levels implies a consistent duality on the other physical levels.

**Fig. 1:**
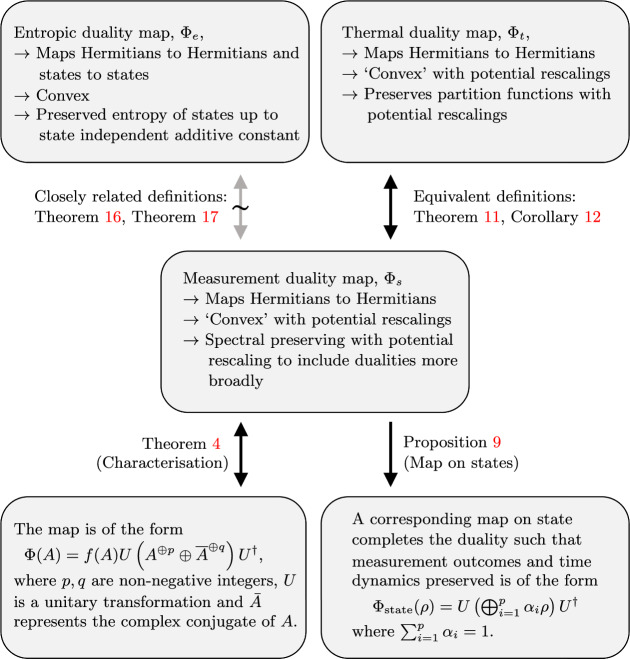
Summary of the main results. We start by considering a duality map $\Phi _s$ that takes in as input observables in one system and outputs the corresponding dual observable in the dual system. There are three constraints that define the map that are be physically motivated, where importantly we allow the map to preserve the eigenspectra (corresponding to measurement outcomes) up to a rescaling—this allows the map to encompass strong–weak dualities. The main contributions consist of: providing a full mathematical characterisation of these generalised maps (Thereom 4) where *f*(*A*) is an operator-dependent rescaling function; showing the form of a consistent map on states is implied by the definition of the operator map Proposition 9; demonstrating the equivalence of thermal dualities and spectral preserving dualities Corollary 12; relating entropic dualities to spectral preserving dualities Theorem 17 to give a new characterisation of entropy preserving maps (see Sect. 3.2.1 for discussion)

The characterisation of entropy preserving maps is a topic of interest independent of simulation with various previous work characterising entropy preserving maps by unitary/antiunitary transformations, [12–14]. Whereas the previous characterisations reduce to Wigner’s theorem, by taking a different route connecting to Jordan and C algebra techniques, we show that allowing an entropic additive constant is precisely the additional freedom that allows the maps to admit a direct sum of both unitary and antiunitary parts. See Fig. 1 for a summary of the results and where the formal statements are found in the paper.

The following section of this paper gives an overview of key previous works related to the theory of analogue simulation. Our generalised definition of a duality map is described and characterised in Sect. 2 with a corresponding map on states. We then show the equivalence of different duality definitions in Sect. 3, highlighting the new characterisation of entropy preserving maps. Finally, we complete the framework by considering errors in the duality map and demonstrating how the framework translates approximate maps to well-controlled errors in physical quantities.

### Previous Work

This work uses some results and techniques from [11] in order to build up a more general framework. This section gives a brief overview of some key results and definitions that are relevant to our investigation, highlighting the constraints that this work will extend.

Encoding maps, denoted $\mathcal {E}$, are at the core of [11]’s simulations. These maps encode all observables, *A*, on the target Hamiltonian system as observables, $A' = \mathcal {E}(A)$, on the simulator Hamiltonian system and are the most restrictive simulations concerning Hamiltonians in a finite-dimensional Hilbert space. The authors give a long list of operational requirements that the encoding map should satisfy to exactly reproduce all physical properties of the target system in the absence of errors:

I Any observable, *A*, on the target system corresponds to an observable on the simulator system so the map must preserve Hermiticity, $\mathcal {E}(A)=\mathcal {E}(A)^\dagger$;
II $\mathcal {E}(A)$ preserves the outcomes, and therefore eigenvalues, of any measurement *A*: $\text {spec}[\mathcal {E}(A)]=\text {spec}[A]$;
III The encoding is real linear, $\mathcal {E}\left( \sum _i \alpha _i h_i \right) = \sum _i \alpha _i \mathcal {E}(h_i)$, for $\alpha _i \in \mathbb {R}$, $h_i \in \text {Herm}$, so that individual Hamiltonian interactions are encoded separately;
IV Measurements are correctly simulated, hence a corresponding map on states, $\mathcal {E}_\text {state}$, should exist such that $\textrm{tr}\left[ \mathcal {E}(A)\mathcal {E}_\text {state}(\rho ) \right] = \textrm{tr}\left[ A \rho \right]$ for all target observables *A*;
V The encoding preserves the partition function up to a physically unimportant constant rescaling (*c*): $Z_{H'}(\beta ) = \textrm{tr}\left[ e^{-\beta \mathcal {E}(H)} \right] = c \textrm{tr}\left[ e^{-\beta H} \right] = c Z_{H}(\beta )$;
VI Time evolution is correctly simulated: $e^{-i\mathcal {E}(H)t}\mathcal {E}_\text {state}(\rho )e^{i\mathcal {E}(H)t} = \mathcal {E}_\text {state}(e^{iHt}\rho e^{iHt})$.

Note the trivial relationships between the physical observables in the simulator and target systems in II–VI, excluding strong–weak dualities. [11] showed that imposing just three operationally motivated conditions on the encoding will necessarily imply that I–VI hold. Furthermore, using Jordan and $C^*$ algebra techniques a mathematical characterisation of encodings was given in the following theorem.^1^

#### Theorem 1

(Characterising encodings; see [11] Theorem 4). An *encoding* map $\mathcal {E}$ from Hermitian $(n\times n)$ matrices ($\textrm{Herm}_n$) to Hermitian $(m \times m)$ matrices satisfies the following constraints for all $A,B\in \textrm{Herm}_n$, and all $p\in [0,1]$:

1. $$\mathcal {E}(A)=\mathcal {E}(A)^\dagger $$
2. $$\textrm{spec}[\mathcal {E}(A)]= \textrm{spec}[A]$$
3. $$\mathcal {E}(pA+(1-p)B)= p \mathcal {E}(A) + (1-p)\mathcal {E}(B)$$

Encodings are necessarily of the form:

1 $$\begin{aligned} \mathcal {E}(M)=U(M^{\oplus p}\oplus \overline{M}^{\oplus q})U^\dagger \end{aligned}$$

for some non-negative integers *p*, *q* and unitary $U\in \mathcal {M}_m$, where $M^{\oplus p}:=\bigoplus _{i=1}^pM$ and $\overline{M}$ denotes complex conjugation.

Note that the operators in the image and domain of the map may act in Hilbert spaces of different dimension (*n* and *m*). Initially no restriction is placed on this. But from the form of the map, it manifests that $m = (p+q)n$ where $(p+q)\ge 1$ so as expected the simulator or dual system is at least as large as the target.

As a consequence of achieving a full mathematical characterisation, it is relatively straightforward to then show that other physical properties are preserved by encodings,

#### Proposition 2

([11] Prop. 28 and discussion). An *encoding* preserves additional physical properties such that there are relationships between:

1. Partition functions, $$\textrm{tr}\left( e^{-\beta \mathcal {E}(H)} \right) = (p+q)\textrm{tr}\left( e^{-\beta H} \right) $$;
2. Entropies, $$S(\mathcal {E}(\rho )) = S(\rho ) + \log (p+q)$$.

Therefore, encodings satisfy condition V without explicitly demanding this as an axiom. There is also a relationship between the entropies of a state and its encoded form which we highlight in Proposition 2.

Preserving the eigenspectra of Hermitian operators hints towards preserving measurement outcomes. However, conditions IV and VI additionally require a corresponding map on states to be well defined. While [11] provide examples of maps on states that, when considered with encodings, give conditions IV and VI, the form of $\mathcal {E}_\textrm{state}$ is not characterised. Note that while the eigenspectra are preserved, the eigenstates of operators including the Hamiltonian may look completely different in the original and encoded case, due to the unitary transformation allowed. However, in particular constructive examples of simulations a close connection between eigenstates can be established, see, e.g. Lemma 20 of [15] and discussion therein. Section 2.2 in fact demonstrates that the form of the map on states is also characterised as an implication of the definition of duality maps on observables and preserving measurement outcomes whereby the state mapping uncomputes this unitary transformation.

An earlier work also posed a definition of simulation based on an isometric encoding map [10]. [11] includes more general maps than simple isometries since anything that satisfies the conditions in Theorem 1 are allowed. [11] also largely restricts to local encodings as the physically relevant case, whereas [10] imposes no formal conditions on the isometry except noting it should be able to be implemented practically.

This framework was altered to consider a simulator system that only reproduces the ground state and first excited state (and hence the spectral gap) of the Hamiltonian, in [16]. The independent interest of gap simulation is demonstrated by applying the framework to the task of Hamiltonian sparsification—exploring the resources required for simplifying the Hamiltonian interaction graph. Aside from the above works, there has been little other follow-up work exploring the theoretical notion of analogue Hamiltonian simulation and duality.

### Motivating Examples

The framework analysed in [11], while the strongest sense of simulation/duality, already encompasses some important cases of physical dualities. For example, fermionic encodings such as the Jordan-Wigner transformation [17, 18] fit into the framework, able to replicate the full physics of the target in the simulator system. Quantum error correcting codes are also examples of ‘simulations in a subspace’ characterised by [11]. In this vein, to begin generalising the current literature we look to physical dualities not yet contained by the current frameworks.

As discussed above, when motivated by duality as opposed to just simulation, an important class is strong–weak dualities. For many of these dualities, there are several aspects that prevent integration with the current frameworks. These challenges can arise due to the absence of a comprehensive mathematical description (such as in the AdS/CFT duality) or the reliance on descriptions involving infinite-dimensional field theories. However, there are simpler instances that still capture some characteristics of strong-dualities while being describable on a finite spin lattice. Here, we describe two examples of strong–weak duality that are closest to our setting which we will use as motivation when extending the current framework.

#### Kramer–Wannier Duality

A paradigmatic example of a strong–weak duality is the Kramer–Wannier duality [1]. Even the isotropic case of this classical duality is not captured by the strong sense of simulation in [11] with the key novel element being the strong–weak nature of the two Hamiltonians. Therefore, this duality was a first benchmark for this generalisation of the theory of simulation to more broadly encompass dualities.

In Kramer–Wannier, an Ising Hamiltonian on a 2d square lattice at high temperature ($\tanh J \beta \ll 1$):

2 $$\begin{aligned} H = - J \sum _{\langle i,j \rangle } \sigma _i \sigma _j, \end{aligned}$$

is dual to another Ising Hamiltonian on the same lattice (in the thermodynamic limit) at low temperature ($\tilde{J}\tilde{\beta }\gg 1$):

3 $$\begin{aligned} \Phi (H) = -\tilde{J} \sum _{\langle i,j \rangle } \sigma _i \sigma _j, \end{aligned}$$

in the thermodynamic limit. The two Hamiltonians are dual, in the sense that their free energies, *f*, are related by

4 $$\begin{aligned} \tilde{\beta }f_{\Phi (H)}= \beta f_H + \ln \sinh (2\beta J), \end{aligned}$$

when the following duality condition relating the interaction strengths and temperature is satisfied:

5 $$\begin{aligned} \tilde{J}\tilde{\beta } = - \frac{1}{2}\ln \tanh (J \beta ). \end{aligned}$$

A more detailed description of this duality and how it arises is given in Appendix E.

This duality can be used to find the critical point for the 2d Ising model since at this point the free energies will be non-analytic. It is in some sense a very simple duality as both Hamiltonians have the same form and act on identical copies of the Hilbert space. However, it follows from the non-trivial nature of the relation between the free energies that expecting all observables to be preserved is too strong. Furthermore, it is clear from the form of the duality that the energy spectrum cannot be preserved without a rescaling. These two aspects of the duality prevent it from fitting into the framework developed in [11].

#### Boson–Fermion Duality

boson–fermion dualities (bosonisation/fermionisation) are a class of dualities transforming between bosonic and fermionic systems, usually in the context of quantum fields. They are an example of particle vortex dualities that have had wide application, particularly in quantum field theory and condensed matter physics. Similarly to the Kramer–Wannier duality, the interest often lies in transforming strongly interacting fermionic systems (e.g. electrons in metals in condensed matter physics) to weakly interacting bosonic systems or vice versa. In particular these dualities often work well near critical points or phase transitions where the crossover of these regimes takes place. In this context, the ‘strong–weak’ nature of the duality can be referred to as a ‘UV to IR’ duality.

There has been extensive study of boson–fermion duality in different dimensions and it is conjectured that an exact duality exists in 3D on the level of partition functions [19–21]. Here, ‘exact’ duality refers to a transformation that is valid in all regimes including at criticality, whereas ‘approximate’ dualities can be demonstrated to hold under some conditions or in specific UV or IR limit. Extending the mathematical framework to also include this type of approximate duality is considered in Sect. 4 where the equivalence is restricted to a subspace, e.g. the low energy subspace. The majority of this paper considers exact mappings, and a duality of this type was demonstrated between 3D lattice gauge theories in [22].

The duality in [22] is between a strongly coupled boson and its free fermion vortex. The bosonic theory is an *XY* model coupled to a *U*(1) Chern–Simons gauge field, where the Chern–Simons theory is realised via a lattice fermion with mass *M* and interaction *U*. The fermionic dual theory is a free massless Dirac fermion implemented by a lattice fermion of mass $M'$ and interaction $U'$. The partition function of the fermionic system is shown to be proportional to the bosonic theory even at criticality, given that the mass and interactions of the two lattice fermions are related via,

6 $$\begin{aligned} \frac{M'}{M} = \frac{I_0(1/T)}{I_1(1/T)}=\sqrt{\frac{1+U'}{1+U}} = {\left\{ \begin{array}{ll} 1 \quad \text {when } T=0,\\ \infty \quad \text {when } T=\infty \end{array}\right. } \end{aligned}$$

where $I_j(x)$ is the *j*th modified Bessel function. The above echoes Eq. (5) giving the duality condition relating the physics of the two systems in different regimes (low and high temperature).

Generally the literature on boson–fermion dualities is out of reach for a duality framework considering operators in finite dimensions as there is a notable gap in our understanding of connecting quantum field theories to finite-dimensional operator algebra. Nevertheless, examining the qualitative aspects of boson–fermion dualities can shed light on deficiencies within the previous mathematical framework. This example reinforces the importance of incorporating non-trivial relationships between spectra to adequately accommodate strong–weak dualities.

## Generalised Duality Map

The first step in studying maps between operators describing a ‘duality’ is to identify what properties these maps should preserve in general. There is potential for wide variation in how duality maps are defined. This work aims for a minimal set of axioms that encompasses as many dualities as possible, in particular strong–weak and high–low-temperature dualities, while capturing [11]’s simulation as a special case. This paper is restricted to consider finite-dimensional systems, we denote Hermitian $(n\times n)$ matrices by $\text {Herm}_n$.

### Definition 3

*(Measurement duality map)*. A *measurement duality map*, $\Phi _s: \textrm{Herm}_n \mapsto \textrm{Herm}_{m}$ satisfies

(i) $$\forall $$ $$a_i\in \textrm{Herm}_n$$, $$p_i\in [0,1]$$ with $$\sum _i p_i = 1:$$
  $$\begin{aligned}\Phi _s\left( \sum _i p_i a_i \right) = G\left( \sum _i p_i a_i \right) \sum _i g( a_i)h(p_i) \Phi _s(a_i);\end{aligned}$$
(ii) $$\forall $$ $$A\in \textrm{Herm}_n:$$
  $$\begin{aligned}\textrm{spec}\left[ \Phi _s(A)\right] = f(A) \textrm{spec}[A].\end{aligned}$$

The scaling functions *f*, *G*, *g*: $\textrm{Herm}_n\mapsto \mathbb {R}$, are Lipschitz on any compact subset of $\textrm{Herm}_n$ and map to zero iff the input is the zero operator. *h*: $[0,1] \mapsto [0,1]$ describes a mapping between probability distributions such that $\sum _i h(p_i) = 1$.

Intuitively, all duality maps must preserve Hermiticity for observables in one theory to be associated with observables in another—this is the most straightforward condition on any duality map. The map is defined to take $(n\times n)$ Hermitian matrices as inputs and output $(m\times m)$ Hermitian matrices. A priori there is no constraint or relation between *n* and *m*, but we will later see as a consequence of the definition that *m*/*n* is a positive integer.

Dualities are also constrained by the convex structure of quantum mechanics, but formulating the minimal requirements in this case is more subtle. Operationally, a convex combination of observables corresponds physically to the process of selecting an observable at random from some ensemble of observables according to some probability distribution, measuring that observable, and reporting the outcome. This is commonly described mathematically by an ensemble of observables: $\{p_i,A_i\}$, where $p_i$ is the probability of measuring observable $A_i$. Since this is a physical operation that can be performed on the original system, there must be a corresponding procedure on the dual system that gives the same outcome. However, this does *not* imply that the dual process must necessarily be given by the convex combination of the dual observables. It would clearly be possible operationally to first rescale the probability distribution before picking the dual observable to measure, and then to rescale the outcome of that measurement in some way before reporting it. A fully general axiomatisation of duality has to allow for this possibility, and this is precisely what is captured mathematically in Axiom (i).^2^

In quantum mechanics, measurement outcomes are associated with the spectra of the Hermitian operators; hence, the final axiom requires a relation between the spectra of dual operators. Again, operationally, we have to allow for the possibility of rescaling the measurement outcomes. Even a simple change of measurement units, which has no *physical* content, induces such a rescaling mathematically. But more general rescalings that interchange large and small eigenvalues are possible, indeed required to encompass strong–weak dualities (e.g. the classic Kramer–Wannier duality).

This is captured mathematically in Axiom (ii) of Definition 3 by the scaling function, *f*, which is observable-dependent. Furthermore, Axiom (ii) imposes a relation on the *set* eigenvalues, but not on their ordering or multiplicities. Thus, which particular dual measurement outcome corresponds to which outcome on the original system can vary. Since the scaling functions depend on the operator, the form of the duality is free to vary for different observables.

The only constraints imposed on the scaling functions *f*, *g*, *G* are those we argue are physically necessary: the range must be restricted to real numbers since all measurement outcomes in quantum mechanics must be real; they are required to satisfy a very weak Lipschitz condition to exclude unphysical discontinuities; and non-vanishing for a nonzero input ensures every observable has a corresponding dual.

There are still plausible notions of duality not captured by this definition. However, the formulation given in Definition 3 is sufficient to restrict to mappings that represent meaningful dualities, yet be a substantial generalisation of Theorem 1.

### Characterisation

A priori, requiring that the spectrum of operators is preserved up to a function that is allowed to depend on the operator itself would appear to be an extremely weak constraint on the map. For example, this function may arbitrarily rescale or invert the spectrum for different operators. However, the interplay between spectrum rescaling and (rescaled) convexity introduces significantly more rigidity into the maps’ structure than either constraint alone. The scaling functions *f*, *g*, *G* appearing in the axioms are found to be necessarily related, such that the axioms can be equivalently rewritten using only a single function. These relationships are proven rather than assumed by initially considering the action of the duality map on orthogonal projectors and proving that the constraints imply a non-trivial preservation of orthogonality (and then building up to general Hermitian operators). A key element of this proof is that intuitively unphysical actions of the map—for example discontinuous permutations within projectors during continuous variations in the operator—can be ruled out using the analyticity of the resolvent of operators at non-degenerate points in its spectrum Appendix D.

#### Theorem 4

(Characterisation). Any *measurement duality map*, $\Phi _s$, with the scale function $f(\cdot )$ is necessarily of the form,

$$\begin{aligned}\Phi _s(A) = f(A) U \left( A^{\oplus p} \oplus \overline{A}^{\oplus q} \right) U^\dagger ,\end{aligned}$$

where *p*, *q* are non-negative integers, *U* is a unitary transformation and $\bar{A}$ represents the complex conjugate of *A*. Equivalently,

$$\begin{aligned}\Phi _s(A) = f(A) U \left( A\otimes P + \overline{A}\otimes Q \right) U^\dagger ,\end{aligned}$$

where *P* and *Q* are orthogonal complemently projectors.

**Fig. 2:**
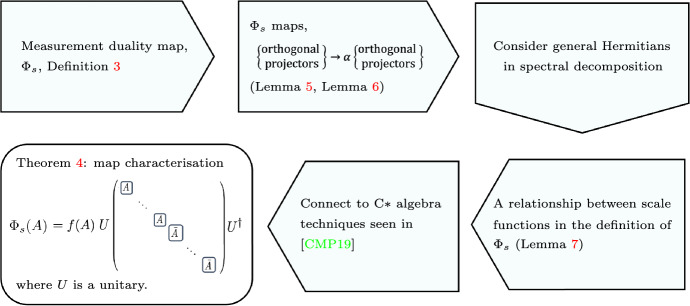
Outline of proof idea for Theorem 4. Starting with the definition of the duality map, we first show that orthogonal projectors are mapped to orthogonal projectors. This then allows one to consider Hamiltonians in their spectral decomposition and map these using the rescaled convexity axiom, there are two technical results as we first show this result for orthogonal complement projectors (Lemma 5) and then general orthogonal projectors (Lemma 6). From consistency, this results in a relationship between the scale functions used to define the map. Substituting these relations into the definition gives new conditions in terms of a single rescaling function *f*. From here, the connection to the characterisation theorem in [11] can be made which leads to the final result: the duality map is necessarily of the form of taking direct sum of copies of the observable and the complex conjugate of the observable, doing a unitary transformation and multiplying by the rescaling function

The rest of this section is dedicated to proving Theorem 4. A sketch of the argument and ingredients used in the proof are outlined in Fig. 2. The result relies on relating duality maps to the encodings characterised in Theorem 1. To demonstrate this, we first need to examine the necessary relations between the different scaling functions which in tern requires establishing how the map transforms orthogonal projectors. The following lemma shows that a duality map will take orthogonal complement projectors to objects proportional to two new orthogonal complement projectors in the new Hilbert space.

#### Lemma 5

(Mapping orthogonal complement projectors). Let $Q_1$ and $Q_2$ be orthogonal complement projectors ($Q_1Q_2 = Q_2Q_1 = 0$ and $Q_1 + Q_2 = \mathbb {I}$). Under a measurement duality map $\Phi _s$, these projectors are mapped to:

$$\begin{aligned}\Phi _s(cQ_1)\propto \Sigma _1 \qquad \Phi _s(cQ_2)\propto \Sigma _2.\end{aligned}$$

Where $c\in \mathbb {R}$ and $\Sigma _1, \Sigma _2$ are themselves orthogonal complement projectors, i.e. $\Sigma _1^2 = \Sigma _1$, $\Sigma _2^2=\Sigma _2$, $\Sigma _1 \Sigma _2 = \Sigma _2 \Sigma _1 = 0$ and $\Sigma _1 + \Sigma _2 = \mathbb {I}$.

#### Proof

Since a general projector $P_i$ has $\text {spec}[P_i]\in \{0,1\}$, by axiom (ii) of Definition 3 the mapped operator has $\text {spec}\left[ \Phi _s(cP_i) \right] = f(cP_i) \text {spec}[cP_i] = cf(cP_i)\text {spec}[P_i]\in cf(cP_i)\{ 0,1 \}$. The map also preserves Hermiticity via definition, so projectors are mapped to operators proportional to projectors. In particular, given orthogonal complement projectors:

7 $$\begin{aligned}&\Phi _s(cQ_1) = cf(cQ_1) \Sigma _1 \end{aligned}$$

8 $$\begin{aligned}&\Phi _s(cQ_2) = cf(cQ_2) \Sigma _2, \end{aligned}$$

it only remains to show that $\Sigma _1,\Sigma _2$ are also orthogonal complement projectors.

The identity is a special case since $\text {spec}[\mathbb {I}] \in \{1 \}$ so $\text {spec}[\Phi _s(\mathbb {I})]\in \{ f(\mathbb {I})\}$. Therefore,

9 $$\begin{aligned} \Phi _s\left( \frac{c}{2}(Q_1 + Q_2)\right) = \Phi _s\left( c\mathbb {I}/2\right) =\frac{c}{2} f\left( c\mathbb {I}/2\right) \mathbb {I} . \end{aligned}$$

Applying axiom (i) to the sum of operators gives,

10 $$\begin{aligned} \Phi _s\left( \frac{c}{2}(Q_1 + Q_2)\right)&= G(c\mathbb {I}/2) h(1/2)\left[ g(cQ_1) \Phi _s(cQ_1) + g(cQ_2)\Phi _s(cQ_2) \right] \end{aligned}$$

11 $$\begin{aligned}&= G(c\mathbb {I}/2)h(1/2)\left[ g(cQ_1)cf(cQ_1)\Sigma _1 + g(Q_2)cf(cQ_2)\Sigma _2 \right] . \end{aligned}$$

Note that while *c* is a general real, (i) has to be applied with $\sum _i p_i = 1$ and $p_i \in [0,1]$, in this case $t_1,t_2 = 1/2$ and *c* has been absorbed into the Hermitian operators.

Equating Eqs. (9) and 11),

12 $$\begin{aligned} G(c\mathbb {I}/2)h(1/2) \left[ g(Q_1)cf(cQ_1)\Sigma _1 + g(Q_2)cf(cQ_2) \Sigma _2 \right] =&\frac{c}{2}f(c\mathbb {I}/2) \mathbb {I} \end{aligned}$$

13 $$\begin{aligned} 2\frac{G(c\mathbb {I}/2)}{f(c\mathbb {I}/2)}h(1/2)\left[ g(cQ_1)f(cQ_1) \Sigma _1 + g(cQ_2)f(cQ_2)\Sigma _2 \right] =&\;\mathbb {I} \end{aligned}$$

14 $$\begin{aligned} \alpha \Sigma _1 + \beta \Sigma _2 =&\;\mathbb {I}, \end{aligned}$$

where the notation is simplified by defining:

15 $$\begin{aligned} \alpha := \frac{2G(c\mathbb {I}/2)h(1/2)g(cQ_1)f(cQ_1)}{f(c\mathbb {I}/2)}, \qquad \beta := \frac{2G(c\mathbb {I}/2)h(1/2)g(cQ_2)f(cQ_2)}{f(c\mathbb {I}/2)}.\nonumber \\ \end{aligned}$$

Rewriting the matrices in Eq. (14) in the $\{\Sigma _1, \Sigma _1^\perp \}$ basis,

16 $$\begin{aligned} \alpha \left( \begin{array}{c|c} \mathbb {I} &{} 0 \\ \hline 0 &{} 0 \end{array} \right) + \beta \left( \begin{array}{c|c} A &{} B \\ \hline C &{} D \end{array} \right) = \left( \begin{array}{c|c} \mathbb {I} &{} 0 \\ \hline 0 &{} \mathbb {I} \end{array} \right) . \end{aligned}$$

Equating the off-diagonal quadrants gives that $\beta B=\beta C = 0$. Since the initial properties of the scaling functions imply that $\beta \ne 0$, *B* and *C* must vanish and $\Sigma _1, \Sigma _2$ are simultaneously diagonalisable with $\left[ \Sigma _1, \Sigma _2\right] =0$. Equating diagonal quadrants gives:

17 $$\begin{aligned}&\alpha \mathbb {I} + \beta A = \mathbb {I} \end{aligned}$$

18 $$\begin{aligned}&\beta D = \mathbb {I}. \end{aligned}$$

In order for $\Sigma _2$ to be a valid projector $D^2=D$ and $A^2=A$. This together with the expression for $D= \frac{1}{\beta } \mathbb {I}$ from Eq. (18) implies that $\beta = +1$ and $D=\mathbb {I}$. Finally, rearranging Eq. (17),

19 $$\begin{aligned} A = (1-\alpha )\mathbb {I} = A^2 = (1-\alpha )^2\mathbb {I}, \end{aligned}$$

together with $\alpha \ne 0$ implies that $\alpha =+1$, $A=0$. In both the above cases, the solutions $\beta =-1$ and $\alpha =-2$ are discarded since $\Sigma _2$ must be a positive definite operator. In the $\{\Sigma _1, \Sigma _1^\perp \}$ basis20so $\Sigma _1,\Sigma _2$ are orthogonal complement projectors. $\square$

The expressions for $\alpha$ and $\beta$ give some initial relations between the scale functions appearing in the axioms:

21 $$\begin{aligned} h(1/2)g(Q_1)f(cQ_1) = h(1/2)g(cQ_2)f(cQ_2) = \frac{f(c\mathbb {I}/2)}{2G(c\mathbb {I}/2)}. \end{aligned}$$

Since for any projector $P_i$ there exists its complement $P_i^\perp$, it follows that the above applies generally for any projector: $h(1/2)g(cP_i)f(cP_i) =\frac{f(c\mathbb {I}/2)}{2\,G(c\mathbb {I}/2)}$.

Now a statement concerning how a measurement duality map acts on two orthogonal projectors that only span a subspace of the initial Hilbert space can be made.

#### Lemma 6

(Mapping orthogonal projectors). Let $P_1$ and $P_2$ be orthogonal projectors such that $P_1P_2 = P_2P_1 = 0$. Under a measurement duality map, $\Phi _s$, these projectors are mapped to:

$$\begin{aligned}\Phi _s(cP_1)\propto \Pi _1 \qquad \Phi _s(cP_2)\propto \Pi _2,\end{aligned}$$

where $c\in \mathbb {R}$ and $\Pi _1, \Pi _2$ are themselves orthogonal projectors.

#### Proof

Again spectrum preservation stipulates that projectors are mapped to objects proportional to projectors:

22 $$\begin{aligned}&\Phi _s(cP_1) = cf(cP_1)\Pi _1 \end{aligned}$$

23 $$\begin{aligned}&\Phi _s(cP_2) = cf(cP_2) \Pi _2 \end{aligned}$$

24 $$\begin{aligned}&\Phi _s\left( \frac{c}{2}(P_1+P_2)\right) = \frac{c}{2}f\left( \frac{c}{2}(P_1 + P_2)\right) \Pi _{12}, \end{aligned}$$

where the final equation holds since the sum of two orthogonal projectors is another projector. Applying axiom (i) to the sum and substituting the above:

25 $$\begin{aligned} \Phi _s\left( \frac{c}{2}(P_1 + P_2)\right)&= G\left( \frac{c}{2}(P_1 + P_2)\right) h(1/2)\left[ g(cP_1)\Phi _s(cP_1) \right. \nonumber \\&\quad \left. + g(cP_2)\Phi _s(cP_2) \right] \end{aligned}$$

26 $$\begin{aligned}&= G\left( \frac{c}{2}(P_1 + P_2)\right) h(1/2)\left[ g(cP_1)cf(cP_1) \Pi _1 \right. \nonumber \\&\quad \left. + g(cP_2)cf(cP_2)\Pi _2 \right] . \end{aligned}$$

Equating Eqs. (24) and (26) in the same way as in Lemma 5 gives:

27 $$\begin{aligned} \alpha (\Pi _1 + \Pi _2 )= \Pi _{12} \end{aligned}$$

where

28 $$\begin{aligned} \alpha&= \frac{2G(c/2(P_1 +P_2))h(1/2)g(cP_1)f(cP_1)}{f(c/2(P_1 + P_2))} \end{aligned}$$

29 $$\begin{aligned}&= \frac{2G(1/2(P_1 + P_2)) h(1/2)g(cP_2)f(cP_2)}{f(c/2(P_1 + P_2))} \end{aligned}$$

30 $$\begin{aligned}&= \frac{G(c/2(P_1+P_2))}{f(c/2(P_1+P_2))}\frac{f(c\mathbb {I}/2)}{G(c\mathbb {I}/2)}. \end{aligned}$$

In the above, the scale factor relation for projectors from Eq. (21) is used to equate $g(cP_1)f(cP_1)=g(cP_2)f(cP_2)$.

Writing the matrices in Eq. (27) in the $\{\Pi _{12}, \Pi _{12}^\perp \}$ basis:31Since $\Pi _1,\Pi _2$ are projectors, they must be positive semi-definite matrices. Let $\left| x\right\rangle$ be a vector only with support on the $\Pi _{12}^\perp$ subspace. The positive semi-definite property requires that3233Only $D=0$ can satisfy the above simultaneously. Once the lower right block is set to 0, the off-diagonal blocks must also vanish for $\Pi _i$ to be valid projectors (see Appendix C),34Therefore, Eq. (27) reduces to the same form as Eq. (16) when examining the top left quadrant only,

35 $$\begin{aligned} \alpha (A_1 + A_2) = \mathbb {I}, \end{aligned}$$

identifying that $\alpha =1$ since $A_1,A_2$ are projectors. Applying Lemma 5 gives $A_1A_2 = A_2A_1 = 0$. The result is that $\Pi _1\Pi _2 = \Pi _2\Pi _1 =0$. $\square$

A consequence of $\alpha =1$ is that,

36 $$\begin{aligned} \frac{G(c/2(P_1+P_2))}{f(c/2(P_1+P_2))} = \frac{G(c\mathbb {I}/2)}{f(c\mathbb {I}/2)}, \end{aligned}$$

for all orthogonal projectors $P_1,P_2$. The above relation can be shown to hold in a more general case which leads to a restatement of the axiom describing the behaviour of the map acting on convex combinations.

#### Lemma 7

(Constrained scale functions). A duality map, $\Phi _s$, satisfies

(i’) $$\Phi _s(\sum _i p_i a_i) = f(\sum _i p_i a_i)\sum _i \frac{p_i}{f(a_i)}\Phi _s(a_i)$$

for all $a_i\in \textrm{Herm}_n$ and $p_i\in [0,1]$ with $\sum _i p_i = 1$.

#### Proof

This proof follows by demonstrating various relationships between the scaling functions *f*, *g*, *G* that must hold as a consequence of Definition 3.

First, for all Hermitian operators *A*, the ratio of *f*(*A*) to *G*(*A*) is proven to be a constant independent of *A*. The spectral decomposition of a general Hermitian operator *A* is given by

37 $$\begin{aligned} A = \sum _i \lambda _i P_i, \end{aligned}$$

where $\lambda _i\in \mathbb {R}$ and in the case of degenerate eigenvalues we are free to chose $\{ P_i\}$ to form a set of orthogonal projectors. In order to apply axiom (i) of Definition 3, the summation is rearranged to read,

38 $$\begin{aligned} A = \sum _i \mu _i \left( c_i P_i \right) , \end{aligned}$$

where now $\mu _i \in [0,1]$ and $\sum _i \mu _i = 1$, whereas $c_i \in \mathbb {R}$ with $\mu _i c_i = \lambda _i$. Note that while clearly this choice of $\mu _i c_i$ is not unique, this does not affect the following argument.

By axiom (i) of Definition 3,

39 $$\begin{aligned} \Phi _s(A) = G(A) \sum _i h(\mu _i) g(c_iP_i)\Phi _s(c_i P_i). \end{aligned}$$

Using Lemma 6, this can be written as a spectral decomposition over orthogonal projectors,

40 $$\begin{aligned} \Phi _s(A) = G(A) \sum _i h(\mu _i) g(c_iP_i) f(c_iP_i)c_i \Pi _i. \end{aligned}$$

However, since the spectral decomposition is unique (up to degenerate eigenvalues where we continue to choose an orthogonal basis) it can also be expressed using the spectrum preserving axiom as

41 $$\begin{aligned} \Phi _s(A) = f(A) \sum _i \mu _ic_i \Pi _{\sigma (i)}, \end{aligned}$$

where $\sigma (i)$ denotes some permutation of indices.

Equating Eqs. (39) and (41) gives,

42 $$\begin{aligned} \frac{G(A)}{f(A)} \sum _i h(\mu _i) g(c_iP_i) f(c_iP_i)c_i \Pi _i= \sum _i \mu _ic_i \Pi _{\sigma (i)}. \end{aligned}$$

Multiplying by $\Pi _k$ selects for a given projector,

43 $$\begin{aligned} \frac{G(A)}{f(A)} h(\mu _{\sigma (j)}) g(c_{\sigma (j)}P_{\sigma (j)}) f(c_{\sigma (j)}P_{\sigma (j)})c_{\sigma (j)} = \mu _jc_j, \end{aligned}$$

where $\sigma (j)=k$. Appendix D demonstrates that in fact $\sigma (k)=k$ $\forall k$ is the only allowed permutation for any map $\Phi _s$ and operator *A*. Therefore, we can equate

44 $$\begin{aligned} h(\mu _i) g( c_iP_i) = \frac{f(A)}{G(A)} \frac{\mu _i}{f(c_iP_i)}. \end{aligned}$$

Since $h(\mu _i) g( c_iP_i)$ cannot depend on the other eigenvalues and vectors of *A* the ratio of *f*(*A*) to *G*(*A*) must be constant for any given Hermitian, i.e.

45 $$\begin{aligned} \frac{f(A)}{G(A)} = x, \qquad \forall A \in \text {Herm}, \end{aligned}$$

for some $$x\in \mathbb {R}$$.

Applying (i) of Definition 3 to the trivial sum ${\Phi _s(A) = G(A)h(t)g(A)\Phi _s(A)}$ gives another useful relation,

46 $$\begin{aligned} g(A) = \frac{1}{G(A)} = \frac{x}{f(A)}, \qquad \forall A \in \text {Herm}, \end{aligned}$$

since $h(1)=1$ by definition.

The next step is to investigate the function *h* by relating *h*(*t*)*g*(*A*) and *g*(*A*). Let $A_1$, $A_2$ be any two Hermitian operators with spectral decompositions,

47 $$\begin{aligned} A_1&= \sum _i \lambda _i P_i\end{aligned}$$

48 $$\begin{aligned} A_2&= \sum _i \mu _i Q_i, \end{aligned}$$

where $\lambda _i,\mu _i \in \mathbb {R}$ such that $\{P_i,Q_i\}$ form an orthogonal set of projectors, i.e. $A_1$ and $A_2$ must have orthogonal support. Consider a convex combination,

49 $$\begin{aligned} A = t A_1 + (1-t)A_2, \end{aligned}$$

with $t \in [0,1]$. Since $A_1$ and $A_2$ have orthogonal support and the map obeys axiom (ii) of Definition 3, the spectrum of the mapped convex combination is:

50 $$\begin{aligned} \text {spec}\left[ \Phi _s(A) \right] =f(A)\{t\lambda _i, (1-t)\mu _i \}. \end{aligned}$$

On the other hand, applying axiom (i) of Definition 3 to *A* gives,

51 $$\begin{aligned} \Phi _s(A) = G(A)\left[ h(t) g(A_1)\Phi _s(A_1) + h(1-t) g(A_2)\Phi _s(A_2) \right] . \end{aligned}$$

By Lemma 6, $\Phi _s(A_1)$ and $\Phi _s(A_2)$ have orthogonal support, and $\{\Phi _s(\lambda _i P_i),\Phi _s(\mu _iQ_i) \}$ is an orthogonal set. Together with axiom (ii) of Definition 3, this implies that

52 $$\begin{aligned} \text {spec}[\Phi _s(A)]&= \{G(A)h(t)g(A_1)\text {spec}[\Phi _s(A_1)],\; G(A)h(1-t)g(A_2)\text {spec}[\Phi _s(A_1)]\} \end{aligned}$$

53 $$\begin{aligned}&= \{G(A)h(t) g(A_1)f(A_1)\lambda _i,\; G(A)h(1-t)g(A_2)f(A_2)\mu _i\}. \end{aligned}$$

Again using the result from Appendix D that the permutation is trivial, we can equate the elements of $\text {spec}\left[ \Phi _s(A) \right]$ that correspond to $A_1$:

54 $$\begin{aligned} f(A)t \lambda _i = G(A)h(t)g(A_1)f(A_1)\lambda _i. \end{aligned}$$

Using Eqs. (45) and (46),

55 $$\begin{aligned} h(t)g(A_1) = \frac{t x}{f(A_1)}, \end{aligned}$$

for all $A_1\in \text {Herm}$ and $t\in [0,1]$.

Finally, substituting for *g*, *G* using Eqs. (45) and (55), (ii) of Definition 3 becomes,

56 $$\begin{aligned} \Phi _s\left( \sum _i p_i A_i \right)&= G\left( \sum _i p_i A_i \right) \sum _i h(p_i)g(A_i)\Phi _s(A_i) \end{aligned}$$

57 $$\begin{aligned}&= \frac{f\left( \sum _i p_i A_i \right) }{x}\sum _i \frac{p_i x}{f(A_i)}\Phi _s(A_i) \end{aligned}$$

58 $$\begin{aligned}&= f\left( \sum _i p_i A_i \right) \sum _i \frac{p_i}{f(A_i)}\Phi _s(A_i) \end{aligned}$$

for all $A_i\in \text {Herm}$ and $p_i \in [0,1]$ where $\sum _i p_i = 1$. $\square$

This constraint on how the map acts on convex combinations of operators enables the link between duality maps and the encodings in Theorem 1 to be made.

#### Proof of Theorem 4

To characterise $\Phi _s$, we define the related map $\mathcal {E}(A): = \frac{\Phi _s(A)}{f(A)}$ and show that $\mathcal {E}$ is an encoding in the sense of Theorem 1. For $\mathcal {E}$ to be an encoding it is sufficient to show that it satisfies the three conditions given in Theorem 1.

Definition 3 states $\Phi _s(A)^\dagger =\Phi _s(A)$, therefore

59 $$\begin{aligned} \mathcal {E}(A)^\dagger = \frac{\Phi _s(A)^\dagger }{\overline{f(A)}} = \frac{\Phi _s(A)}{\overline{f(A)}}. \end{aligned}$$

However, $\overline{f(A)} = f(A)$ since it is defined be a real function. Therefore, $\mathcal {E}(A)^\dagger = \mathcal {E}(A)$ and the first encoding axiom is satisfied.

Using (ii) of Definition 3, it quickly follows that $\mathcal {E}$ is spectrum preserving:

60 $$\begin{aligned} \text {spec}\left[ \mathcal {E}(A)\right]&= \text {spec} \left[ \frac{\Phi _s(A)}{f(A)} \right] \end{aligned}$$

61 $$\begin{aligned}&= \frac{1}{f(A)}\text {spec}\left[ \Phi _s(A)\right] \end{aligned}$$

62 $$\begin{aligned}&= \frac{1}{f(A)} f(A)\text {spec}[A] \end{aligned}$$

63 $$\begin{aligned}&= \text {spec}[A]. \end{aligned}$$

The final encoding axiom is shown using (i) of Definition 3 and Lemma 7 to demonstrate that $\mathcal {E}$ is convex,

64 $$\begin{aligned} \mathcal {E}\left( \sum _i p_i a_i\right)&= \frac{\Phi _s(\sum _i p_i a_i)}{f(\sum _i p_i a_i)} \end{aligned}$$

65 $$\begin{aligned}&= \frac{1}{f(\sum _i p_i a_i)}f\left( \sum _i p_i a_i\right) \sum _i \frac{p_i}{f(a_i)}\Phi _s(a_i) \end{aligned}$$

66 $$\begin{aligned}&= \sum _i \frac{p_i}{f(a_i)} f(a_i)\mathcal {E}(a_i) \end{aligned}$$

67 $$\begin{aligned}&= \sum _i p_i \mathcal {E}(a_i). \end{aligned}$$

The mathematical form follows directly from $\Phi _s(A)=f(A)\mathcal {E}(A)$ and Theorem 1. $\square$

### Map on States

A map on Hamiltonians and observables is not enough to fully characterise the duality, since a state in one theory should also have a corresponding state in the other. The set of states is just a subset of Hermitian operators; however, the physical requirements on the state map differ to those given in Definition 3. Instead, when we consider maps on states, we need them to be compatible with the map on operators such that measurement outcomes and time dynamics behave as expected. In the following definition, we use $\mathcal {H}_n$ to denote a Hilbert space of dimension $(n\times n)$ and $\mathcal {S}(\mathcal {H})$ to denote the set of states in Hilbert space $\mathcal {H}$.

#### Definition 8

(Compatible duality state map) Given a duality map, $\Phi$, on operators (Definition 3), we say that a map on states, $\Phi _\textrm{state}: \mathcal {S}(\mathcal {H}_n)\mapsto \mathcal {S}(\mathcal {H}_m)$, is compatible with $\Phi$ if it satisfies the following properties:

1. Convexity: for all ${p_i\in [0,1]}$ and ${\sum _i p_i = 1}$,
  $$\begin{aligned} {\Phi _\textrm{state}\left( \sum _i p_i\rho _i\right) = \sum _i p_i\Phi _\textrm{state}(\rho _i)};\end{aligned}$$
2. Measurement outcomes are preserved up to the scaling function,
  $$\begin{aligned} \textrm{tr}\left[ \Phi (A)\Phi _\textrm{state}(\rho ) \right] = f(A) \textrm{tr}\left[ A \rho \right] \end{aligned}$$
  for all $$A\in \textrm{Herm}_n$$, $$\rho \in \mathcal {S}(\mathcal {H}_n)$$;
3. Time dynamics is consistent at rescaled times,
  $$\begin{aligned} \Phi _\textrm{state}\left( e^{-iHt}\rho e^{iHt} \right) = e^{-i\Phi (H)t/f(H)} \Phi _\textrm{state}(\rho ) e^{i\Phi (H)t/f(H)}.\end{aligned}$$

While examples of compatible maps on states were given for the simulations in [11], this section proves that the form of the map on states is implied by the definitions of duality maps and the corresponding map on states.

#### Proposition 9

(Form of state map) Given a duality map, $\Phi (A) = f(A) U \left( \bigoplus _{i=1}^p A \oplus \bigoplus _{i=p+1}^{p+q} \bar{A}\right) U^\dagger$, on operators, the compatible duality map on states, $\Phi _\textrm{state}: \mathcal {S}(\mathcal {H}_n)\mapsto \mathcal {S}(\mathcal {H}_m)$, as in Definition 8, is necessarily of the form:

$$\begin{aligned}\Phi _\textrm{state}(\rho ) = U\left( \bigoplus _{i=1}^{p}\alpha _i \rho \right) U^\dagger ,\end{aligned}$$

where $$\alpha _i \in [0,1]$$ and $$\sum _{i=1}^p \alpha _i= 1$$.

#### Proof

Setting $B = e^{iHt}$ and conjugating condition 3 of compatible duality state maps with $U^\dagger$

68 $$\begin{aligned} U^\dagger \Phi _\text {state}\left( B \rho B^\dagger \right) U&= U^\dagger e^{i\Phi (H)t/f(H)}\Phi _\text {state}(\rho )e^{-\Phi (H)t/f(H)}U \end{aligned}$$

69 $$\begin{aligned}&= \left( B^{\oplus p}\oplus \bar{B}^{\oplus q} \right) U^\dagger \Phi _\text {state}(\rho ) U \left( (B^\dagger )^{\oplus p}\oplus (\bar{B}^\dagger )^{\oplus q} \right) . \end{aligned}$$

Since *B* represents time evolution for general *t* and *H*, the above shows that the conjugated state map must have the same block diagonal structure as $\Phi$, i.e.

70 $$\begin{aligned} U^\dagger \Phi _\text {state}(\rho )U = \bigoplus _{i=1}^{p+q}X_i (\rho ). \end{aligned}$$

We now substitute this structure of the state map into condition 2 of the definition of compatible state maps:

71 $$\begin{aligned} \textrm{tr}(A\rho )&= \textrm{tr}\left[ U \left( \bigoplus _{i=1}^p A \oplus \bigoplus _{i=p+1}^{p+q} \bar{A} \right) U^\dagger U \bigoplus _{i=1}^{p+q} X_i(\rho )U^\dagger \right] \end{aligned}$$

72 $$\begin{aligned}&= \textrm{tr}\left[ \bigoplus _{i=1}^p A X_i(\rho ) \oplus \bigoplus _{i=p+1}^{p+q} \bar{A}X_i(\rho ) \right] \end{aligned}$$

73 $$\begin{aligned}&= \sum _{i=1}^{p}\textrm{tr}\left[ AX_i(\rho ) \right] + \sum _{i=p+1}^q \textrm{tr}\left[ \bar{A}X_i(\rho ) \right] . \end{aligned}$$

Since Eq. (73) is true for all *A* we can differentiate with respect to *A*,

74 $$\begin{aligned} \rho = \sum _{i=1}^p X_i(\rho ), \end{aligned}$$

and separately with respect to $\bar{A}$,

75 $$\begin{aligned} 0 = \sum _{i=p+1}^{p+q} X_i(\rho ). \end{aligned}$$

Note that *A* and $\bar{A}$ are independent for the purpose of differentiation.

The fact that $\Phi _\text {state}$ maps states to states implies that $X_i(\rho )$ is a positive operator for all *i* and $\rho \in \mathcal {S}(\mathcal {H}_n)$. Apply $X_i$ to a pure state $\left| \psi _0\right\rangle$ and assume for contradiction that the image has some support on a distinct pure state which wlog we call $\left| \psi _1\right\rangle$,

76 $$\begin{aligned} X_i\left( \left| \psi _0\right\rangle \left\langle \psi _0\right| \right) = \alpha _i \left| \psi _0\right\rangle \left\langle \psi _0\right| + \beta _i \left| \psi _1\right\rangle \left\langle \psi _1\right| + \text {else}, \end{aligned}$$

where “else” has no overlap with $\left| \psi _0\right\rangle$ or $\left| \psi _1\right\rangle$. $0 \le \alpha _i,\beta _i \le 1$ since $X_i(\rho )$ is a positive operator. From Eq. (74),

77 $$\begin{aligned} \left| \psi _0\right\rangle \left\langle \psi _0\right|&= \sum _{i=1}^p X_i (\left| \psi _0\right\rangle \left\langle \psi _0\right| ) \end{aligned}$$

78 $$\begin{aligned}&= \sum _{i=1}^p \alpha _i \left| \psi _0\right\rangle \left\langle \psi _0\right| + \beta _i \left| \psi _1\right\rangle \left\langle \psi _1\right| + \text {else}. \end{aligned}$$

Therefore, $\sum _{i=1}^p \alpha _i = 1$ and $\sum _{i=1}^{p}\beta _i = 0$ $\implies$ $\beta _i = 0$ for all *i*. Hence when applied to any pure state each $X_i$ for $i\in [1,p]$ acts as,

79 $$\begin{aligned} X_i(\left| \psi \right\rangle \left\langle \psi \right| ) = \alpha _i \left| \psi \right\rangle \left\langle \psi \right| \qquad \text {with} \qquad \sum _{i=1}^p \alpha _i = 1. \end{aligned}$$

It follows from condition 1 that each $X_i$ is individually convex. Explicitly

80 $$\begin{aligned} U \bigoplus _{i=1}^{p+q}X_i\left( \sum _j t_j \rho _j\right) U^\dagger = \sum _j t_j U \oplus _{i=1}^{p+q}X_i(\rho _j)U^\dagger \end{aligned}$$

implies that for all *i* the following is true

81 $$\begin{aligned} X_i \left( \sum _j t_j \rho _j\right) = \sum _j t_j X_i (\rho _j). \end{aligned}$$

This combined with Eq. (79) gives for any state $\rho \in \mathcal {S}(\mathcal {H}_n)$,

82 $$\begin{aligned} X_i(\rho ) = \alpha _i \rho \qquad \text {with} \qquad \sum _{i=1}^p \alpha _i = 1. \end{aligned}$$

By normalisation, $X_i(\rho ) = 0$ for $i\in [p+1,q]$ which can also be seen from Eq. (75) by applying a similar argument as for $b_i=0$.

Equation (82) combined with Eq. (70) gives the quoted form of the map.

$$\square $$

## Equivalent Definitions of Duality

Similarly to Proposition 2, once we establish the characterisation of the measurement duality map, it becomes clear that other physical properties are necessarily related in the dual systems, in particular the partition functions and entropies. Certain dualities, such as Bosonisation and Kramer–Wannier, are imposed on the level of partition functions. Therefore, while the measurement duality maps are candidates to describe these types of dualities, this one-way implication does not preclude other mathematical mappings that preserve thermal properties and describe these dual phenomena.

This section establishes the reverse equivalence: duality definitions based on the preservation of partition functions or entropies are in fact essentially equivalent to the measurement duality maps defined in the previous section. This connection is particularly interesting to unify different dualities on the level of partition functions, measurement outcomes and entropies.

The connection between partition functions and the spectra arises from a transformation of a partition function equality into an infinite sequence of polynomials in the charges (e.g. $\beta$ for the Hamiltonian). This sequence is shown to converge in the limit to a relation between the $\ell _\infty$ norms of the spectra. A recursive application of this argument then implies the preservation of the spectral sets themselves. The connection between entropy preserving and spectrum preserving is perhaps more surprising, and leads to a novel result concerning the characterisation of entropy preserving maps up to an additive constant.

### Partition Function Duality

Examples of physical dualities suggest that it is common for a duality to be defined in terms of partition functions (or equivalently free energy), rather than observables, particularly when considering classical thermodynamics. This motivates considering a different definition of duality, formulated in terms of preserving partition functions rather than measurement outcomes:

#### Definition 10

(Thermal duality map). A *thermal duality map*, $\Phi _t: \textrm{Herm}_n \mapsto \textrm{Herm}_{m}$ satisfies

(i) $$\forall $$ $$a_i\in \textrm{Herm}_n$$, $$p_i\in [0,1]$$ with $$\sum _i p_i = 1:$$
  $$\begin{aligned}\Phi _t\left( \sum _i p_i a_i \right) = G\left( \sum _i p_i a_i \right) \sum _i g( a_i)h(p_i) \Phi _t(a_i);\end{aligned}$$
(ii) $\forall$ $A\in \textrm{Herm}_n$ and all $J_A>0$, $J_A\in \mathbb {R}$:
  $$\begin{aligned}\alpha \textrm{tr}\left[ e^{-J_Af(A)A} \right] = \textrm{tr}\left[ e^{-J_A\Phi _t(A)}\right] \end{aligned}$$
  for some constant $\alpha >0$.

The scaling functions *f*, *G*, *g*: $\textrm{Herm}_n\mapsto \mathbb {R}$, are Lipschitz on any compact subset of $\textrm{Herm}_n$ and map to zero iff the input is the zero operator. However, *h*: $[0,1] \mapsto [0,1]$ where $\sum _i h(p_i) = 1$ iff $\sum _i p_i = 1$.

The convexity condition is the same as in Definition 3, as is the motivation. The second axiom captures how the thermal physics of the two systems are related. The simplest physical example of this is the Hamiltonian of the system, *H*, with inverse temperature, $\beta$, acting as the corresponding charge $J_H$. However, if the duality is to be complete, this relationship should also hold for other source terms in the partition function $\textrm{tr}\left[ -\beta H + \sum _i J_{A_i}A_i \right]$ to relate both the thermal properties and correlations of the two systems. We must again allow the freedom of rescaling the values of the charges in the dual system by an operator-dependent scaling function *f*, since this is something that could be done operationally. Equating these generalised partition functions for all values of the charges is mathematically equivalent to (ii), since trivially all but one selected charge can be set to 0 in tern.

The following result demonstrates that a map preserving partition functions up to a physical rescaling as in Definition 10 necessarily preserved the spectra up to the same rescaling.

#### Theorem 11

(Maps preserving partition functions preserve spectra). Given a map $\Phi _t: \textrm{Herm}_n \mapsto \textrm{Herm}_{m}$ such that $\forall$ $A\in \textrm{Herm}_n$ and all $J_A>0$, $J_A\in \mathbb {R}$

$$\begin{aligned}\alpha \textrm{tr}\left[ e^{-J_Af(A)A} \right] = \textrm{tr}\left[ e^{-J_A\Phi _t(A)}\right] \text { for some constant } \alpha >0,\end{aligned}$$

where *f* :  $\textrm{Herm}_n\mapsto \mathbb {R}$ is a scaling factor. $\forall$ $A\in \textrm{Herm}_n$, $\Phi _t$ satisfies,

$$\begin{aligned}\textrm{spec}\left[ \Phi _t(A)\right] = f(A) \textrm{spec}[A].\end{aligned}$$

#### Proof

Initially let $\text {spec}\left[ A \right] = \{\lambda _i \}$ and $\text {spec}\left[ \Phi _t(A)\right] = \{\mu _i \}$ and relate their ”partition functions” as in the theorem statement

83 $$\begin{aligned} \alpha \sum _i e^{-J f(A)\lambda _i} = \alpha \textrm{tr}\left[ e^{-J f(A)A} \right] =&\textrm{tr}\left[ e^{-J \Phi _t(A)} \right] =\sum _j e^{-J \mu _j}. \end{aligned}$$

Expanding the exponential using the Maclaurin series, $e^x = \sum _{k=0}^\infty \frac{x^k}{k!}$, which converges for all *x*, gives

84 $$\begin{aligned} \alpha \sum _i^{\dim [A] } \sum _{k=0}^\infty \frac{(-J f(A)\lambda _i)^k}{k!} = \sum _j^{\dim \left[ \Phi _t(A)\right] } \sum _{k=0}^\infty \frac{(-J \mu _j)^k}{k!}. \end{aligned}$$

For the above polynomials to be equal at all values of the charge *J*, the coefficients for each power of *J* must be equal.^3^ Equating the $J^0$ coefficients fixes the relationship between the dimensions:

85 $$\begin{aligned} \alpha \dim [A ]= \dim \left[ \Phi _t(A)\right] . \end{aligned}$$

Therefore, the operators *A* and $\Phi _t(A)$ may act on Hilbert spaces of different dimension (i.e. $n\ne m$). However, Eq. (85) implies that $\alpha$ is a positive rational so we set $\frac{x}{y}:=\alpha$ with $x,y\in \mathbb {Z}^+$ coprime in the following.

For a given *A*, the remaining equalities generate an infinite system of polynomials in $\{\mu _i \}_{i=1}^{\dim \left[ \Phi _t(A)\right] }$,

$$\begin{aligned} \forall p \in \mathbb {Z}^+ \nonumber :\; \frac{x}{y} \sum _{i=1}^{ \dim [A ]} \left( f(A)\lambda _i\right) ^p = \sum _{i=1}^{\dim \left[ \Phi _t(A)\right] } \mu _i^p. \qquad \end{aligned}$$

Manipulating the sum to remove the multiplicative factors we have $\forall p \in \mathbb {Z}^+$,

86 $$\begin{aligned} \sum _{i=1}^{ x\dim [A ]} \left( f(A)\lambda '_i \right) ^p = \sum _{i=1}^{y\dim \left[ \Phi _t(A)\right] } \mu '^p_i, \end{aligned}$$

where we define new vectors $\lambda '$, $\mu '$ with elements $\{\lambda '_{(i-1)x+n}\}_{n=1}^{x} = \lambda _i$ and $\{\mu '_{(i-1)y+n}\}_{n=1}^{y} = \mu _i$, indexing the elements of all vectors in non-decreasing order.

The summations in Eq. (86) now each contain the same number of terms and thus, for even $p=2\varrho$, we can interpret the above as equating the *p*-norms of two $(x\dim [A ] = y\dim \left[ \Phi _t(A)\right] )$-dimensional vectors:

87 $$\begin{aligned} \left( \sum _{i=1}^{ x\dim [A]} |f(A)\lambda '_i |^{2\varrho }\right) ^{1/2\varrho } = \left( \sum _{i=1}^{y\dim [\Phi _t(A)]} |\mu '_i |^{2\varrho }\right) ^{1/2\varrho }. \end{aligned}$$

Taking the limit $\varrho \rightarrow \infty$, this converges to the $\ell _\infty$ norm of both sides, i.e. we can equate the elements of maximum absolute value in each vector: $\max _i |f(A)\lambda _i' |= \max _i |\mu _i' |$.

Now, subtracting $(\max _i f(A)\lambda _i')^{2\varrho } = (\max _i\mu _i')^{2\varrho }$ from both sides of Eq. (87), we obtain an analogous set of *p*-norm equalities but for vectors with length reduced by 1, with the maximum elements removed. Applying this argument recursively, we conclude that the vectors $f(A)\lambda '$ and $\mu '$ must have identical components up to signs.

The linear variant of Eq. (86) rules out the case where the components $\lambda '$ and $\mu '$ have different signs:

88 $$\begin{aligned} \sum _{i=1}^{ x\dim [A ]} f(A)\lambda '_i = \sum _{i=1}^{y\dim \left[ \Phi _t(A)\right] } \mu '_i = \sum _{i=1}^{x\dim [A ]} \pm f(A)\lambda '_i. \end{aligned}$$

This follows as Eq. (83) must hold for all Hermitians *A*, including those with only positive eigenvalues. Any term in the sum being negated on the right hand side of Eq. (88) would produce a strictly smaller total than that of the left hand side; therefore,

89 $$\begin{aligned} \mu ' = f(A)\lambda '. \end{aligned}$$

It remains to use $\lambda '$ and $\mu '$ to find the relation between the original eigenvalue vectors $\lambda$ and $\mu$ (potentially of different lengths). Choose an *A* with non-degenerate spectrum, and consider the two smallest eigenvalues of *A*. We have

90 $$\begin{aligned}&\lambda _1 = \lambda '_{x} = \frac{\mu '_{x}}{f(A)} \end{aligned}$$

91 $$\begin{aligned}&\lambda _2= \lambda '_{x+1} = \frac{\mu '_{x+1}}{f(A)}. \end{aligned}$$

Since *A* has non-degenerate spectrum, we have $\mu '_{x}\ne \mu '_{x+1}$. But $\{\mu '_{(i-1)y+n}\}_{n=1}^{y}$ are equal for all *i* by definition of $\mu '$. Thus, $x\ge y$ and $y=1$, since *x* and *y* are coprime. Hence, $\dim \Phi _t(A)$ must be at least as large as $\dim A$ and $\alpha \in \mathbb {Z}^+$.

Equation (89) and $\alpha \in \mathbb {Z}^+$ implies the set equality $\{\mu _i\}_{i=1}^{\alpha \dim [A]}=f(A)\{\lambda _i\}_{i=1}^{\dim [A]}$, where each element of $\mu$ is alpha-fold degenerate. The two spectra are thus proportional and the proof is complete. $\square$

A simple corollary of this result is that the thermal duality map defined above is equivalent to the previously studied and characterised measurement duality map:

#### Corollary 12

The set of maps that describe a thermal duality is equal to the set of maps describing a measurement duality, such that Definitions 3 and 10 are equivalent. Therefore, thermal duality maps are also of the form,

$$\begin{aligned} \Phi _t(A) = f(A) U \left( A^{\oplus p} \oplus \overline{A}^{\oplus q} \right) U^\dagger ,\end{aligned}$$

where *p*, *q* are non-negative integers, *U* is a unitary transformation and $\bar{A}$ represents the complex conjugate of *A*.

#### Proof

Recall that a measurement duality map is defined by two conditions

(I.) $$\Phi _s \left( \sum _i p_i a_i \right) = G\left( \sum _i p_i a_i \right) \sum _i g(a_i)h(p_i)\Phi _s(a_i)$$;
(II.) $$\textrm{spec}[\Phi _s(A)] = f(A) \textrm{spec}[A]$$.

and a partition function duality is also defined by two conditions,

(i.) $$\Phi _t \left( \sum _i p_i a_i \right) = f\left( \sum _i p_i a_i \right) \sum _i \frac{p_i}{f(a_i)}\Phi _t(a_i)$$;
(ii.) $$\alpha \textrm{tr}\left[ e^{-J_Af(A)A} \right] = \textrm{tr}\left[ e^{-J_A\Phi _t(A)}\right] \text { for some constant } \alpha >0$$.

(I) and (i) are identical statements. From Theorem 11, (ii) implies (II) where the degeneracy of the spectrum is given by $\alpha$ which was shown to necessarily be $\alpha \in \mathbb {Z}^+$. The reverse implication (II) implies (ii) can be shown. Given the measurement duality characterisation, the spectrum is not only equal, but each eigenvalue has degeneracy $p+q = m/n \in \mathbb {Z}^+$. Therefore,

92 $$\begin{aligned} \textrm{tr}\left[ e^{-J_A\Phi _s(A)}\right] = \frac{m}{n}\textrm{tr}\left[ e^{-J_Af(A)A}\right] , \end{aligned}$$

where we can equate $\alpha = m/n$.

All of the conditions have been shown to be equivalent; therefore, the two definitions of duality describe the same set of maps. $\square$

### Entropic Duality

A third and final viewpoint is to consider entropic dualities.

#### Definition 13

(Entropic duality map) An *entropic duality map*, $\Phi _e: \textrm{Herm}_n \mapsto \textrm{Herm}_{\alpha n}$ and $\Phi _e: \mathcal {S}(\mathcal {H}_n)\mapsto \mathcal {S}(\mathcal {H}_{\alpha n})$ satisfies

(i) $$\forall $$ $$a_i\in \textrm{Herm}_n$$, $$p_i\in [0,1]$$ with $$\sum _i p_i = 1:$$
  $$\begin{aligned}\Phi _e\left( \sum _i p_i a_i \right) = \sum _i p_i \Phi _e(a_i);\end{aligned}$$
(ii) $$\forall \rho \in \mathcal {S}(\mathcal {H}):$$
  $$\begin{aligned}S(\Phi _e(\rho )) = S(\rho ) + \log \alpha ;\end{aligned}$$
(iii) $$\Phi _e(0)=0$$.

The justification for the convexity condition is unchanged. However, the map is additionally constrained to map states to states (positive operators with unit trace) to meaningfully examine the behaviour of dual entropies. An immediate consequence of this is a simplification of the previously allowed generalised convexity to standard convexity.

The second axiom captures how the entropies of corresponding states are related. In trivial examples of dual states in different sized spaces, there is additional entropy arising from the additional degrees of freedom in the larger state space. This gives an additive offset that depends on the Hilbert space dimension in the entropy relation. For example, if states $\rho$ are mapped to the (trivially) dual states $\Phi (\rho ) = \rho \otimes 1/d$, the entropy of the dual state picks up an additional additive contribution: $S(\Phi (\rho )) = S(\rho ) + d$.

More generally, for a $d_1$-dimensional maximally mixed state to be dual to the maximally mixed state in $d_2>d_1$ dimensions, the required entropy relation is

93 $$\begin{aligned} S\left( \frac{1}{d_2}\mathbb {I}_{(d_2\times d_2)} \right)&= \log d_2 \end{aligned}$$

94 $$\begin{aligned}&= \log \frac{d_2 d_1}{d_1} \end{aligned}$$

95 $$\begin{aligned}&= S\left( \frac{1}{d_1}\mathbb {I}_{(d_1\times d_1)} \right) + \log \frac{d_2}{d_1}. \end{aligned}$$

Then, $\alpha = d_2/d_1$ and we can identify $\log \alpha$ as a constant entropy offset arising from the different Hilbert space dimensions.

Entropies in quantum information theory express the information content or entanglement of systems. For example, in holographic dualities such as AdS/CFT there are relationships between the entropy of corresponding states (the Ryu–Takayanagi formula [24]). However, the above definition concerns the global entropy of states and not entanglement entropy of reduced states. Therefore, the *state dependent* additive entropy that appears in the Ryu–Takayanagi formula does not contradict the *state independent* additive entropy we assert, since the latter does not refer to the entropy of a reduced state but rather a state on the full Hilbert space.

Similarly to the previous section, we arrive at a characterisation of entropic duality maps by demonstrating that a map that preserves entropies is necessarily spectrum preserving. To show this result, we first need some technical lemmas.

#### Lemma 14

(Entropy of mixtures of mixed states). Given a density operator, $\rho _\mathcal {A} = \sum _{x=1}^k p_x \rho _x$, that is a probabilistic mixture of mixed states $\rho _x$, with $p_x\in [0,1]$ and $\sum _x p_x =1$. The von Neumann entropy of $\rho _\mathcal {A}$ obeys the following equality,

$$\begin{aligned}S(\rho _\mathcal {A}) = \sum _x p_x S(\rho _x) -\sum _x p_x \log p_x,\end{aligned}$$

if and only if $\rho _x$ have orthogonal support, i.e. $\textrm{tr}[\rho _x \rho _y]=0$ for all $x\ne y$.

#### Lemma 15

(Pure states mapped to orthogonal density matrices). Let $\{ \sigma _i \}_{i=1}^d$ be a set of orthogonal pure states that forms a basis in $\mathcal {H}$, with $\sigma _i \in P_1(\mathcal {H})$. Let the map $\phi : \mathcal {S}(\mathcal {H}_n)\mapsto \mathcal {S}(\mathcal {H}_{\alpha n})$, be

- Entropy preserving up to an additive constant, $S(\phi (\rho )) = S(\rho ) + \log \alpha$;
- Convex, $\phi (t\rho + (1-t)\sigma ) = t \phi (\rho ) + (1-t) \phi (\sigma )$. However, $t\in [0,1]$ and $\rho ,\sigma \in \mathcal {S}(\mathcal {H})$.

The image of this set under the map is a new set, $\{\phi (\sigma _i) \}_{i=1}^d$, with orthogonal support.

For the proof of Lemma 14 (Lemma 15), see Appendix A (Appendix B), respectively. Now that an orthogonal basis in the dual system is established, we can show that the entropy preserving map is necessarily spectrum preserving. Not that in this case the map is only transforming between states, how the map acts on the full Hermitians is the subject of the next result.

#### Theorem 16

(Entropy preserving implies spectrum preserving on positive normalised Hermitian operators). A map $\phi : \mathcal {S}(\mathcal {H}_n)\mapsto \mathcal {S}(\mathcal {H}_{\alpha n})$, that is

- Entropy preserving up to an additive constant: $S(\phi (\rho )) = S(\rho ) + \log \alpha$;
- Convex: $\phi (t\rho + (1-t)\sigma ) = t \phi (\rho ) + (1-t) \phi (\sigma )$. Where $t\in [0,1]$ and $\rho ,\sigma \in \mathcal {S}(\mathcal {H})$

will transform the spectrum of the density operator in the following way

$$\begin{aligned}&\textrm{spec}[\rho ] = \{\lambda _1, ...,\lambda _d \}\\&\textrm{spec}\left[ \phi (\rho )\right] = \left\{ \frac{\lambda _1}{\alpha },...,\frac{\lambda _d}{\alpha } \right\} \end{aligned}$$

where every eigenvalue in the spectrum of $\phi (\rho )$ has multiplicity $\alpha$.

#### Proof

The first step in the proof is to show that the image of the pure states $\{\phi (\sigma _i)\}_{i=1}^d$—which by Lemma 15 is known to have orthogonal support—has $\alpha$ nonzero eigenvalues all equal to $1/\alpha$. Using the entropy preserving property of the map: $S(\phi (\sigma _i))=\log \alpha$. Since $\log \alpha$ is the maximal entropy of a Hilbert space of dimension $\alpha$, it follows that $\phi (\sigma _i)$ must have at least $\alpha$ nonzero eigenvalues, i.e. $\text {Rank}\left[ \phi (\sigma _i)\right] \ge \alpha$ for all *i*.

As a consequence of orthogonality, the rank summation of *d* mixed states, $\phi (\sigma _i)$, will be upper bounded by the dimension of the Hilbert space the density matrices act in:

96 $$\begin{aligned} \sum _{i=1}^d \text {Rank} \left[ \phi (\sigma _i) \right] \le \alpha d. \end{aligned}$$

It follows that $\text {Rank}\left[ \phi (\sigma _i)\right] = \alpha$ for all *i*. Together with the entropy $S(\phi (\sigma _i))=\log \alpha$, it follows that the nonzero eigenvalues must be flat and $\text {spec}\left[ \phi (\sigma _i) \right] = \{1/\alpha ,0 \}$.

It is then simple to extend to the full result. Any state in $\rho \in \mathcal {S}(\mathcal {H})$ can be written as a linear combination of pure states $\rho = \sum _{i=1}^d \lambda _i \sigma _i$ where due to normalisation $\sum _{i=1}^d \lambda _i = 1$. Using the convexity property of the map

97 $$\begin{aligned} \phi \left( \sum _{i=1}^d \lambda _i \sigma _i \right) = \sum _{i=1}^d \lambda _i \phi (\sigma _i). \end{aligned}$$

From Lemma 14, $\{ \phi (\sigma _i)\}$ have orthogonal support and therefore $\text {spec}\left[ \phi (\sigma _i) \right] = \{1/\alpha ,0 \}$. Therefore, the spectrum of $\phi (\rho )$ will be $\{\lambda _1/\alpha , \lambda _2/\alpha ,..., \lambda _d/\alpha \}$ each with multiplicity $\alpha$. $\square$

Armed with a link between entropy preserving and spectral preserving on positive Hermitians with unit trace, we can now look to characterising the entropic dual maps on the full Hermitian space. We show that the entropic definition of duality is only slightly less general than the others. This originates from the normalisation of elements of $\mathcal {S}(\mathcal {H})$ whereby since the operator map the is restricted to map states to states the rescaling is limited. The following result characterised entropic duality maps and describes the almost equivalence to the two other types of duality map we have studied.

#### Theorem 17

Every entropic duality map $\Phi _e$ is a measurement/thermal duality map where $f(A)=1/\alpha$ for all $A\in \textrm{Herm}_n$ and therefore has the form

$$\begin{aligned}\Phi _e(A) = \frac{1}{\alpha }U \left( A^{\oplus p} \oplus \overline{A}^{\oplus q} \right) U^\dagger ,\end{aligned}$$

for some unitary *U* and $p,q\in \mathbb {Z}^+$. Conversely if $\Phi$ is a measurement/thermal duality map then the related map

$$\begin{aligned} \Phi '_e(A):= {\left\{ \begin{array}{ll} \frac{\Phi (A)}{\alpha f(A)} &{} \textrm{for }\quad A\in \textrm{Herm}_n \ne 0\\ \Phi (A) &{} \textrm{for }\quad A=0, \end{array}\right. } \end{aligned}$$

is an entropic duality map.

#### Proof

Corollary 12 states that measurement and thermal duality maps are equivalent. Therefore, this proof can focus on demonstrating a relationship between $\Phi _e$ and measurement duality maps and the connection to thermal duality maps in identical.

Recall that an entropic duality map is defined by three conditions

(i.) $$\Phi _e\left( \sum _i p_i a_i \right) = \sum _i p_i \Phi _e(a_i)$$;
(ii.) $$S(\Phi _e(\rho )) = S(\rho ) + \log \alpha $$;
(iii.) $$\Phi _e(0)=0$$;

and a measurement duality map is defined by two conditions

(I.) $$\Phi _s \left( \sum _i p_i a_i \right) = f\left( \sum _i p_i a_i \right) \sum _i \frac{p_i}{f(a_i)}\Phi _s(a_i)$$;
(II.) $$\textrm{spec}[\Phi _s(A)] = f(A) \textrm{spec}[A]$$.

We have used Lemma 7 to replace the original weakened convexity condition with the constrained convexity condition that equivalently defines the map. Additionally, restricting to the case where $f(A) = \frac{1}{\alpha }$ then condition I becomes,

98 $$\begin{aligned} \Phi _s\left( \sum _i p_i a_i\right) = \frac{1}{\alpha } \sum \alpha p_i \Phi _s(a_i) = \sum _i p_i \Phi _s(a_i) \end{aligned}$$

such that it is manifestly equivalent to (i) for this choice of scale function.

All that is left to do for the first statement is to show that a map obeying (i)-(iii) is spectrum preserving for all Hermitians. The first step is to show that (i) & (iii) implies the map, $\Phi _e$, is real linear. This follows from the same argument laid out in the proof of [11] Theorem 4. For any real negative $\lambda$ set $p=\frac{\lambda }{\lambda -1}>0$, $A\in \text {Herm}_n$ and $B = \frac{pA}{(p-1)}= \lambda A$. Using (i) and (iii) together:

99 $$\begin{aligned} \Phi _e(pA + (1-p)B)&= \Phi _e(0) = 0 \end{aligned}$$

100 $$\begin{aligned}&= p \Phi _e(A) + (1-p)\Phi _e(\lambda A). \end{aligned}$$

Therefore $\lambda \Phi _e(A) = \Phi _e(\lambda A)$. Repeating this logic for $\lambda A$ gives $\lambda ^2 \Phi _e(A) = \Phi _e(\lambda ^2 A)$ and hence homogeneity for all real scalars. Then combining (i) with homogeneity gives real linearity of $\Phi _e$, i.e.

101 $$\begin{aligned} \Phi _e\left( \sum _i p_i \lambda a_i\right)&= \sum _i p_i \Phi _e(\lambda a_i) = \sum _i \lambda p_i a_i, \end{aligned}$$

for $$(\lambda p_i)\in \mathbb {R}$$ and $$a_i\in \text {Herm}_n$$.

The entropic duality map restricted to $\mathcal {S}(\mathcal {H}_n)$ satisfies the conditions of Theorem 16 and therefore $\Phi _e$ preserves the spectra of positive Hermitians with unit trace (up to a renormalisation). The transformation of the spectra of $M\not \in \mathcal {S}(\mathcal {H})$ by $\Phi _e$ is shown by building up from $\sigma ,\rho \in \mathcal {S}(\mathcal {H})$ using $\Phi _e(a\rho +b\sigma )= a \Phi _e(\rho ) + b \Phi _e(\sigma )$. First note that any Hermitian operator can be written in a spectral decomposition $M = \sum _i \nu _i \left| \psi _i\right\rangle \left\langle \psi _i\right|$. Splitting the decomposition up into two sums over the positive and negative eigenvalues, respectively,

102 $$\begin{aligned} M&= \sum _{\nu _i>0}\nu _i \left| \psi _i\right\rangle \left\langle \psi _i\right| + \sum _{\nu _i<0}\nu _i \left| \psi _i\right\rangle \left\langle \psi _i\right| \end{aligned}$$

103 $$\begin{aligned}&= M_+ + M_- \end{aligned}$$

104 $$\begin{aligned}&= c_+ \rho _+ + c_- \rho _-, \end{aligned}$$

where $\rho _{+/-} = \frac{M_{+/-}}{\textrm{tr}(M_{+/-})}$ and $c_{+/-}=\textrm{tr}(M_{+/-})$. Therefore

105 $$\begin{aligned} \Phi _e(M) = c_+ \Phi _e(\rho _+) + c_- \Phi _e(\rho _-). \end{aligned}$$

Since $\rho _+$ and $\rho _-$ are orthogonal it follows from Theorem 16 and Lemma 15 that the spectrum of *M*, $\{ \nu _i\}_{i=1}^d$ transforms as

106 $$\begin{aligned} \textrm{spec}\left[ \Phi _e(M)\right] =\frac{1}{\alpha } \left\{ \nu _1,...,\nu _d\right\} , \end{aligned}$$

where every eigenvalue in the new spectrum has multiplicity $\alpha$.

The converse statement is simple to demonstrate. For all $A\in \textrm{Herm}_n$, $\Phi (A) = \Phi (A)^\dagger$ and since $f(A)\in \mathbb {R}$ it follows that $\Phi '_e$ also preserves Hermiticity. Using the simplified convexity axiom from Lemma 7 for $\Phi$, and substituting for $\Phi '_e$, it is easy to see that this map is convex as in (i) of the definition of entropic duality maps. Finally using spectrum preservation of $\Phi$,

107 $$\begin{aligned} \text {spec}\left[ \Phi '_e(\rho ) \right] = \frac{1}{\alpha } \text {spec}\left[ \rho \right] , \end{aligned}$$

for a state $\rho \in \text {Herm}_n$, where each eigenvalue has $\alpha$ copies. $S(\rho ) = \sum _i \eta _i \log \eta _i$ where $\{ \eta _i\}$ are the eigenvalues of $\rho$. Therefore, the entropy of the mapped state is,

108 $$\begin{aligned} S(\Phi '_e(\rho ))&= -\alpha \sum _i \left( \frac{\eta _i}{\alpha } \right) \log \left( \frac{\eta _i}{\alpha } \right) \end{aligned}$$

109 $$\begin{aligned}&= - \sum _i \eta _i \log \eta _i + \sum _i \eta _i \log \alpha \end{aligned}$$

110 $$\begin{aligned}&= S(\rho ) + \log \alpha , \end{aligned}$$

and the second axiom of Definition 13 is satisfied by the map. The third axiom follows immediately from $\Phi '_e(0):= \Phi (0)=0$, giving the converse statement.

$$\square $$

#### Extension to Wigner’s Theorem: A New Characterisation of Entropy Preserving Maps

The above connection between entropy preserving and spectrum preserving axioms is notable since there is independent interest in characterising entropy preserving maps. While it is well known that a unitary or anitunitary^4^ transformation leaves the entropy invariant, the reverse implication is false without additional information. Previous work, that traces its origins back to Wigner’s celebrated theorem [25], has shown that by demanding additional constraints on entropy preserving maps, the maps are entirely characterised by either a unitary or antiunitary transformation.

##### Proposition 18

(Previous entropic map characterisations). Given a surjective map on states $\phi : \mathcal {S}(\mathcal {H})\mapsto \mathcal {S}(\mathcal {H})$ where the Hilbert space $\mathcal {H}$ has dimension *n*,

1. [13] For all $$\rho \in \mathcal {S}(\mathcal {H})$$, $$\forall \lambda \in [0,1]$$
  $$\begin{aligned}S(\lambda \rho + (1-\lambda )\textrm{1}/n) = S(\lambda \phi (\rho ) + (1-\lambda )\textrm{1}/n)\end{aligned}$$
  iff $\phi (\rho ) = W \rho W^*$ for some unitary or antiunitary operator *W*.
2. [14] For all $$\rho ,\sigma \in \mathcal {S}(\mathcal {H})$$, $$\forall \lambda \in [0,1]$$
  $$\begin{aligned}S(\rho + (1-\lambda )\sigma ) = S(\lambda \phi (\rho ) + (1-\lambda )\phi (\sigma ))\end{aligned}$$
  iff $\phi (\rho ) = W \rho W^*$ for some unitary or antiunitary operator *W*.
3. [12] For all $$\rho ,\sigma \in \mathcal {S}(\mathcal {H})$$,
  $$\begin{aligned}S(\rho ||\sigma ) = S(\phi (\rho )||\phi (\sigma ))\end{aligned}$$
  iff $\phi (\rho ) = W \rho W^*$ for some unitary or antiunitary operator *W*.

These can be translated into the language used in our characterisation theorem by noting that for any antiunitary operator *W*, the operator *WK*, where *K* is the complex conjugation operator, is unitary. Therefore, in Proposition 18 either *W* is unitary which corresponds to $p=1$, $q=0$ or if *W* is antiunitary, for some unitary *U*, $\phi (\rho ) = U \overline{\rho } U^\dagger$ corresponding to $p=0$, $q=1$. Hence, all maps in the above proposition are found to be encodings with $p+q\le 1$.

However, to our knowledge maps preserving entropy up to an additive constant have not been studied in the literature. A direct consequence of Theorem 17 is a natural extension of these previous generalisations of Wigner’s theorem arises. A map $\Phi : \mathcal {S}(\mathcal {H})\mapsto \mathcal {S}(\mathcal {H}^{\oplus \alpha })$ is convex,

111 $$\begin{aligned} \Phi \left( \sum _i p_i \rho _i\right) = \sum _i p_i \Phi (\rho _i), \end{aligned}$$

and entropy preserving up to an additive constant

112 $$\begin{aligned} S(\Phi (\rho _i)) = S(\rho _i) + \log \alpha , \end{aligned}$$

for all $\rho _i \in \mathcal {S}(\mathcal {H})$, $p_i \in [0,1]$ with $\sum _i p_i = 1$, where $\alpha \in \mathbb {Z}_{\ge 0}$; iff $\Phi$ is of the form,

113 $$\begin{aligned} \Phi (\rho ) = U \left( \bigoplus _{i=1}^p V_i \rho V_i^\dagger \oplus \bigoplus _{i=p+1}^{p+q}W_i\rho _i W_i^\dagger \right) U^\dagger \end{aligned}$$

for some unitaries $U,V_i$ and antiunitaries $W_i$ acting on $\mathcal {H}$, where $p,q\in \mathbb {Z}_{\ge 0}$ and $p+q = \alpha$.

Whereas previous characterisations of entropy preserving maps reduce to Wigner’s theorem, by taking a different route via Jordan and $C^*$ algebra techniques we show that the entropic additive constant is precisely the additional freedom that allows the maps to admit a direct sum of both unitary and antiunitary parts.

## Approximate Dualities

So far only exact dualities have been considered. However, more general definitions of duality are needed in order for this framework to be practical. This section defines how to extend the ideas of exact duality maps to allow for approximations and restrictions to a subspace. Here, approximate refers to the physics of the two systems being equal up to some error, the approximate equivalence holds within the full subspace. However, the simulation within a subspace corresponds to the other type of ‘approximate’ duality discussed in the Bosonisation example, where the equivalence only holds in some regime, e.g. the low energy regime.

### Definition 19

($(\mathcal {S}, \epsilon )$-Duality). $\tilde{\Phi }$: $\textrm{Herm}_n\mapsto \textrm{Herm}_m$ is a $(\mathcal {S}, \epsilon )$-approximate duality map if $\exists$ a duality map $\Phi$ such that $\forall A\in \textrm{Herm}_n$, the action of $\tilde{\Phi }$ restricted to the subspace $\mathcal {S}$ is close to the action of $\Phi$:

$$\begin{aligned}\Vert \left. \tilde{\Phi }(A)\right| _{\mathcal {S}} - \Phi (A)\Vert \le k(A) \epsilon ,\end{aligned}$$

for some constant $\epsilon$, where $k:\textrm{Herm}_n\mapsto \mathbb {R}_{\ge 0}.$ The duality map is:

(i.) **exact** if $$\epsilon =0$$;
(ii.) **unital** if $f(A)=1$ for all $A\in \textrm{Herm}_n$.

[11] places a large emphasis on local simulations given the focus on Hamiltonian simulation. Since many-body Hamiltonians of interest are often local, a local encoding will preserve this local structure. Exact dualities by simple extension are those related to a local encoding,

### Definition 20

(Local duality map). A local duality map $\Phi : \textrm{Herm}_n \mapsto \textrm{Herm}_m$ is a duality map i.e. of the form $\Phi (A) = f(A)\mathcal {E}(A)$, where the corresponding encoding $\mathcal {E}$ is a local encoding in the sense of [11] definition 13.

Due to the close relation between duality maps and encodings, we can extend the above definition to focus on approximately local duality maps.

### Definition 21

($(\mathcal {S}, \epsilon ,\eta )$-Local duality). $\tilde{\Phi }$: $\textrm{Herm}_n\mapsto \textrm{Herm}_m$ is a $(\mathcal {S}, \epsilon , \eta )$-approximately local duality map if it is an $(\mathcal {S}, \epsilon )$-approximate duality map and the exact duality map $\Phi (M) = f(M)V \left( M^{\oplus p}\oplus \overline{M}^{\oplus q} \right) V^\dagger$ in Definition 19 is close to a local duality map (Definition 20), $\Phi '(M) = f(M)V' \left( M^{\oplus p}\oplus \overline{M}^{\oplus q} \right) V'^\dagger$, such that $\Vert V - V'\Vert \le \eta$. The duality is **exactly-local** if $\eta =0$.

Locality is a natural property to consider, but similar definitions could be equivalently formulated for some other desirable properties, for example, particle number conserving. How these error parameters translate to errors in the physically relevant properties is explored in Sect. 4.3.

The remainder of this section demonstrates that the definition of duality mappings (and their approximate counterparts), arising from physically motivated axioms, have several desirable properties. In particular, exact and approximate dualities are shown to compose well. The choice of extension to approximate mappings is further motivated since the errors defined are shown propagate to physically relevant properties in a controlled way.

### Similar Mappings

As expected, if two exact duality maps are close the results of applying the maps to the same operator are also close. Furthermore, applying the same mapping to two close operators gives outputs that are close. This was formalised for encodings in Lemma 19 of [11], here we show a similar result for duality maps where, unsurprisingly, the “closeness” now also depends on the scaling functions of the maps involved.

First we restate Lemma 18 of [11], a technical result used in the following proof.

#### Lemma 22

Let $A,B: \mathcal {H}\rightarrow \mathcal {H}'$ and $C:\mathcal {H}\rightarrow \mathcal {H}$ be linear maps. Let $\Vert \cdot \Vert _a$ be the trace or operator norm. Then,

114 $$\begin{aligned} \Vert ACA^\dagger - BCB^\dagger \Vert _a\le (\Vert A\Vert +\Vert B\Vert )\Vert A-B\Vert \hspace{2pt} \Vert C\Vert _a. \end{aligned}$$

#### Proposition 23

(Similar exact dualities). Consider two duality maps $\Phi$ and $\Phi '$ defined by ${\Phi (M) = f(M)V\left( M^{\oplus p}\oplus \overline{M}^{\oplus q} \right) V^\dagger }$, $\Phi '(M)=f'(M)V' \left( M^{\oplus p}\oplus \overline{M}^{\oplus q} \right) V'^\dagger$, for some isometries *V*, $V'$. Then for any operators *M* and $M'$:

(i) $$\Vert \Phi (M) - \Phi '(M)\Vert \le \left( |\sqrt{f(M)} | + |\sqrt{f'(M)} | \right) \Vert \sqrt{f(M)}V - \sqrt{f'(M)}V' \Vert \Vert M\Vert $$;
(ii) $$\Vert \Phi (M)-\Phi (M')\Vert = \Vert f(M)M-f(M')M'\Vert $$.

#### Proof

For *(i)* applying Lemma 22 gives

115 $$\begin{aligned} \Vert \Phi (M)-\Phi '(M)\Vert&=\Vert f(M)V\textbf{M}V^\dagger - f'(M)V'\textbf{M}V'^\dagger \Vert \end{aligned}$$

116 $$\begin{aligned}&\le \left( \Vert \sqrt{f(A)}V\Vert + \Vert \sqrt{f'(M)}V'\Vert \right) \Vert \sqrt{f(M)}V - \sqrt{f'(M)}V'\Vert \Vert M\Vert \end{aligned}$$

117 $$\begin{aligned}&= \left( |\sqrt{f(M)} |+ |\sqrt{f'(M)} | \right) \Vert \sqrt{f(M)}V - \sqrt{f'(M)}V'\Vert \Vert M\Vert , \end{aligned}$$

where $\textbf{M}=M^{\oplus p}\oplus \overline{M}^{\oplus q}$. The second part is simply

118 $$\begin{aligned} \Vert \Phi (M) - \Phi (M')\Vert&= \Vert f(M)V\left( M^{\oplus p} \oplus \overline{M}^{\oplus q} \right) V^\dagger - f(M')V\left( M'^{\oplus p} \oplus \overline{M'}^{\oplus q} \right) V^\dagger \Vert \end{aligned}$$

119 $$\begin{aligned}&= \Vert f(M)V\left( \left( M-\frac{f(M')}{f(M)}M'\right) ^{\oplus p} \oplus \left( \overline{M}-\frac{f(M')}{f(M)}\overline{M'}\right) ^{\oplus q} \right) V^\dagger \Vert \end{aligned}$$

120 $$\begin{aligned}&= \Vert f(M)M-f(M')M'\Vert . \end{aligned}$$

$$\square $$

### Composition

It follows almost directly from [11] Lemma 17 that the composition of two exact duality maps, $\Phi = \Phi _2 \circ \Phi _1$, will itself be an exact duality map; therefore, we first restate their result.

#### Lemma 24

If $\mathcal {E}_1$ and $\mathcal {E}_2$ are encodings, then their composition $\mathcal {E}_1\circ \mathcal {E}_2$ is also an encoding, Furthermore, if $\mathcal {E}_1$ and $\mathcal {E}_2$ are both local, then their composition $\mathcal {E}_1\circ \mathcal {E}_2$ is local.

#### Proposition 25

(Exact duality map composition). Let $\Phi _1$ and $\Phi _2$ be duality maps. The composition of these maps, $\Phi = \Phi _2 \circ \Phi _1$, is also a duality map with the valid duality scaling function $f(\cdot )=f_2(\Phi _1(\cdot ))f_1(\cdot )$. Furthermore, if the initial dualities were both local, the composition is also local.

#### Proof

The two duality maps necessarily have the form

121 $$\begin{aligned} \Phi _1(M)&= f_1(M)V_1 \left( M\otimes P_1 + \overline{M}\otimes Q_1\right) V_1^\dagger , \end{aligned}$$

122 $$\begin{aligned} \Phi _2(M)&= f_2(M)V_2 \left( M\otimes P_2 + \overline{M}\otimes Q_2\right) V_2^\dagger , \end{aligned}$$

where $V_i$ are isometries, $f_i$ are real functions, and $P_i$, $Q_i$ are orthogonal projectors. This leads to a composition of the form,

123 $$\begin{aligned} \! (\Phi _2 \circ \Phi _1)(M)= & {} f_2(f_1(M)V_1 \left( M\otimes P_1 + \overline{M} \otimes Q_1\right) V_1^\dagger )f_1(M) \times \nonumber \\{} & {} V_2 \left[ V_1 \left( M\otimes P_1 + \overline{M}\otimes Q_1\right) V_1^\dagger \otimes P_1 \right. \nonumber \\{} & {} + \left. \overline{V_1 \left( M\otimes P_2 +\overline{M}\otimes Q_2\right) V_1^\dagger }\otimes Q_1\right] V_2^\dagger . \end{aligned}$$

Lemma 24 tells us this can be rewritten as,

124 $$\begin{aligned} (\Phi _2 \circ \Phi _1)(M)&= f_2(f_1(M)V_1 \left( M\otimes P_1 + \overline{M}\otimes Q_1\right) V_1^\dagger ) f_1(M)\times \nonumber \\&\quad U\left[ M \otimes P + \overline{M}\otimes Q \right] U^\dagger , \end{aligned}$$

where $U=V_2(V_1 \otimes P_2+ \overline{V_1}\otimes Q_2 + \mathbb {I}\otimes (\mathbb {I}- P_2-Q_2))V_2^\dagger$ is an isometry and $P=P_1\otimes P_2+ \overline{Q}_1\otimes Q_2$, $Q=Q_1\otimes P_2+\overline{P}_1\otimes Q_2$ are new orthogonal projectors.

All that remains is to identify a new scaling function,

125 $$\begin{aligned} f(M) = f_2(f_1(M)V_1 \left( M\otimes P_1 + \overline{M}\otimes Q_1\right) V_1^\dagger )f_1(M), \end{aligned}$$

and note that it satisfies the three prerequisites from the definition of a duality map. The first two are immediate: it maps operators to real scalars and does not map to zero unless the operator is zero. Checking the function is also Lipschitz on compact sets requires slightly more work.

We would like to show for all $B,B'$ in any compact subset there exists a constant *L* such that,

126 $$\begin{aligned} |f_2(\Phi _1(B))f_1(B) - f_2(\Phi _1(B'))f_1(B') | \le L \Vert B-B'\Vert . \end{aligned}$$

Breaking this down and using knowledge of $f_1,f_2$,

127 $$\begin{aligned}&|f_2(\Phi _1(B))f_1(B) - f_2(\Phi _1(B'))f_1(B') | \nonumber \\&\le |f_2(\Phi _1(B)) ||f_1(B)-f_1(B') | + |f_1(B') ||f_2(\Phi _1(B))-f_2(\Phi _1(B')) |\end{aligned}$$

128 $$\begin{aligned}&\le |f_2(\Phi _1(B)) |L_1 \Vert B-B'\Vert +|f_1(B') |L_2 \Vert \Phi _1(B)-\Phi _1(B')\Vert . \end{aligned}$$

Using result (ii) from Proposition 23,

129 $$\begin{aligned} \Vert \Phi _1(B)-\Phi _1(B')\Vert&\le |f_1(B') |\Vert B-B'\Vert + |f_1(B)-f_1(B') |\Vert B\Vert \end{aligned}$$

130 $$\begin{aligned}&\le |f_1(B') |\Vert B-B'\Vert + L_1\Vert B\Vert \Vert B-B'\Vert . \end{aligned}$$

Therefore,

131 $$\begin{aligned}&|f_2(\Phi _1(B))f_1(B) - f_2(\Phi _1(B'))f_1(B') | \nonumber \\&\le \left( |f_2(\Phi _1(B)) | L_1 + |f_1(B') |L_2 \left( |f_1(B') |+L_1\Vert B\Vert \right) \right) \Vert B-B'\Vert \end{aligned}$$

132 $$\begin{aligned}&\le L \Vert B-B'\Vert . \end{aligned}$$

The function is then a valid rescaling since for all *B*, *B* in a compact set there exists a constant *L* such that,

133 $$\begin{aligned} |f_2(\Phi _1(B)) | L_1 + |f_1(B') |L_2 \left( |f_1(B') |+L_1\Vert B\Vert \right) \le L, \end{aligned}$$

as compactness implies $\Vert B\Vert ,f_2(\Phi _1(B)),f_1(B)$ can be upper bounded by a constant.

The scale factor is independent of the locality structure so it follows directly from Lemma 24 that if the initial dualities were both local the composition is also local. $\square$

This can now be extended to consider how the error parameters translate when two approximately local duality maps are composed.

#### Proposition 26

(Approximate duality composition). Let $\tilde{\Phi }_1$, $\tilde{\Phi }_2$ be $(\mathcal {S}_i, \epsilon _i, \eta _i)$-approximately local duality maps with corresponding close exact duality maps $\Phi _1(\cdot )=f_1(\cdot )\mathcal {E}_1(\cdot )$, $\Phi _2=f_2(\cdot )\mathcal {E}_2(\cdot )$, respectively. Their composition $\tilde{\Phi } = \tilde{\Phi }_2\circ \tilde{\Phi }_1$ is a $(\mathcal {S}, \epsilon , \eta )$-approximately local duality map on any compact subset where,

134 $$\begin{aligned}&\epsilon = \epsilon _1 + \epsilon _2, \end{aligned}$$

135 $$\begin{aligned}&\eta \le \eta _1 + \eta _2, \end{aligned}$$

136 $$\begin{aligned}&k(A) = k_2\left( \tilde{\Phi }_1\left. (A)\right| _{\mathcal {S}_1}\right) + L_2k_1(A)^2\epsilon _1 \nonumber \\&\qquad \qquad + \Lambda _2 |f_1(A) |\Vert A\Vert k_1(A)+|f_2(\Phi _1(A)) |k_1(A). \end{aligned}$$

Here, $L_2$ is the Lipschitz constant of $f_2$. Moreover, the exact duality that is close to the approximate composition is the composition of exact dualities, $\Phi _2\circ \Phi _1$. $\mathcal {S}\subseteq \mathcal {S}_2$ is the subspace given by the domain of $\Phi _2$ when the range is restricted to $\mathcal {S}_1$.

#### Proof

Since $\tilde{\Phi }_1$ and $\tilde{\Phi }_2$ are approximate dualities,

137 $$\begin{aligned}&\left\| \left. \tilde{\Phi }_1(A)\right| _{\mathcal {S}_1}- \Phi _1(A)\right\| \le k_1(A)\epsilon _1 \end{aligned}$$

138 $$\begin{aligned}&\left\| \left. \tilde{\Phi }_2(A)\right| _{\mathcal {S}_2}- \Phi _2(A)\right\| \le k_2(A)\epsilon _2. \end{aligned}$$

For $\tilde{\Phi }$ to be an approximate duality, it must satisfy an inequality of the following form,

139 $$\begin{aligned} \left\| \left. \tilde{\Phi }_2\left( \left. \tilde{\Phi }_1(A)\right| _{\mathcal {S}_1}\right) \right| _{\mathcal {S}_2}- \Phi (A)\right\| \le k(A)\epsilon , \end{aligned}$$

for some exact duality $\Phi$, where we have used knowledge of $\mathcal {S}$ to rewrite the restriction. Exact dualities compose to give a valid exact duality $\Phi _2\circ \Phi _1(A) = f_2(\Phi _1(A))f_1(A)\mathcal {E}_2\circ \mathcal {E}_1(A)$ (see Proposition 25). So we take this as $\Phi$ in Eq. (139) and show that the norm difference is bounded by something of the form of the right hand side of Eq. (139).

Using the knowledge of the composite dualities and the triangle inequality,

140 $$\begin{aligned}{} & {} \left\| \tilde{\Phi }_2\left. \left( \tilde{\Phi }_1\left. (A)\right| _{\mathcal {S}_1} \right) \right| _{\mathcal {S}_2} - \Phi _2\circ \Phi _1(A)\right\| \nonumber \\{} & {} \quad \le k_2\left( \tilde{\Phi }_1\left. (A)\right| _{\mathcal {S}_1}\right) \epsilon _2 + \left\| \Phi _2\left( \tilde{\Phi }_1\left. (A)\right| _{\mathcal {S}_1}\right) - \Phi _2\circ \Phi _1(A)\right\| . \end{aligned}$$

The second term in Eq. (140) can be broken down using the similar exact dualities result (ii) from Proposition 23,

141 $$\begin{aligned}&\Vert \Phi _2\left( \tilde{\Phi }_1\left. (A)\right| _{\mathcal {S}_1}\right) - \Phi _2\circ \Phi _1(A)\Vert \nonumber \\&\quad = \Vert f_2(\tilde{\Phi }_1\left. (A)\right| _{\mathcal {S}_1})\tilde{\Phi }_1\left. (A)\right| _{\mathcal {S}_1} - f_2(\Phi _1(A))\Phi _1(A)\Vert \end{aligned}$$

142 $$\begin{aligned}&\quad \le \Bigl |\Bigl |\left( f_2(\tilde{\Phi }_1\left. (A)\right| _{\mathcal {S}_1})-f_2(\Phi _1(A))+f_2(\Phi _1(A))\right) \left( \tilde{\Phi }_1\left. (A)\right| _{\mathcal {S}_1}- \Phi _1(A)\right. \nonumber \\&\qquad \quad \left. +\Phi _1(A)\right) - f_2(\Phi _1(A))\tilde{\Phi }_1\left. (A)\right| _{\mathcal {S}_1}\Bigl |\Bigl | \end{aligned}$$

143 $$\begin{aligned}&\quad \le |f_2(\tilde{\Phi }_1\left. (A)\right| _{\mathcal {S}_1})-f_2(\Phi _1(A)) |\left( \Vert \tilde{\Phi }_1\left. (A)\right| _{\mathcal {S}_1}- \Phi _1(A)\Vert +\Vert \Phi _1(A)\Vert \right) \nonumber \\&\qquad +|f_2(\Phi _1(A)) |\Vert \tilde{\Phi }_1\left. (A)\right| _{\mathcal {S}_1}-\Phi _1(A)\Vert \end{aligned}$$

144 $$\begin{aligned}&\quad \le |f_2(\tilde{\Phi }_1\left. (A)\right| _{\mathcal {S}_1})-f_2(\Phi _1(A)) |\left( k_1(A)\epsilon _1+|f_1(A) |\Vert A\Vert \right) \nonumber \\&\qquad + |f_2(\Phi _1(A)) |k_1(A)\epsilon _1. \end{aligned}$$

Substituting this back gives,

145 $$\begin{aligned}{} & {} \Vert \tilde{\Phi }_2\left. \left( \tilde{\Phi }_1\left. (A)\right| _{\mathcal {S}_1}\right) \right| _{\mathcal {S}_2} - \Phi _2\circ \Phi _1(A)\Vert \nonumber \\{} & {} \quad \le k_2\left( \tilde{\Phi }_1\left. (A)\right| _{\mathcal {S}_1}\right) \epsilon _2 + |f_2(\tilde{\Phi }_1\left. (A)\right| _{\mathcal {S}_1})-f_2(\Phi _1(A)) |\nonumber \\{} & {} \qquad \times \Big (k_1(A)\epsilon _1+|f_1(A) |\Vert A\Vert \Big ) + |f_2(\Phi _1(A)) |k_1(A)\epsilon _1. \end{aligned}$$

Since $f_2$ is Lipschitz on any compact subset,

146 $$\begin{aligned}{} & {} \Vert \tilde{\Phi }_2\left. \left( \tilde{\Phi }_1\left. (A)\right| _{\mathcal {S}_1}\right) \right| _{\mathcal {S}_2} - \Phi _2\circ \Phi _1(A)\Vert \nonumber \\{} & {} \quad \le k_2\left( \tilde{\Phi }_1\left. (A)\right| _{\mathcal {S}_1}\right) \epsilon _2 + L_2 k_1(A)\epsilon _1\Big (k_1(A)\epsilon _1 +|f_1(A) |\Vert A\Vert \Big )\nonumber \\{} & {} \qquad + |f_2(\Phi _1(A)) |k_1(A)\epsilon _1, \end{aligned}$$

and all terms on the right hand size are of order $\epsilon _1$ or $\epsilon _2$. One choice of $\epsilon$ and *k*(*A*) is then,

147 $$\begin{aligned} \epsilon&= \epsilon _1+\epsilon _2 \end{aligned}$$

148 $$\begin{aligned} k(A)&= k_2\left( \tilde{\Phi }_1\left. (A)\right| _{\mathcal {S}_1}\right) + L_2k_1(A)^2\epsilon _1 \nonumber \\&\quad + L_2 |f_1(A) |\Vert A\Vert k_1(A)+|f_2(\Phi _1(A)) |k_1(A). \end{aligned}$$

The scaling of $\eta$ is simplified by the definition of the subspace $\mathcal {S}$, since $\Phi _1$/$\Phi _2$ are $\eta _1/\eta _2$ close to local dualities $\Phi _1'/\Phi _2'$. Therefore by Lemma 24 and triangle inequality, we have $\Vert V-V'\Vert \le \eta _1 + \eta _2$. $\square$

### Physical Properties

This section walks through how the parameters in the definition of approximate and approximately local duality translates to different physical properties.

#### Measurement Outcomes

Definition 3 includes a spectrum preserving statement motivated by considering that dual measurement outcomes should be related. This included a scaling factor relating the spectra which is associated with a possible unit rescaling. Now considering approximate duality maps, the rescaled eigenvalues of corresponding observables are approximately equal with a controlled error.

##### Proposition 27

(Approximate eigenvalues). Let the Hermitian operator *A* act on $\left( \mathbb {C}^d \right) ^{\otimes n}$ and $\tilde{\Phi }$ be a $(\mathcal {S},\epsilon ,\eta )$- approximately local duality map. Let $\lambda _i(A)$, $\lambda _i(\tilde{\Phi }(A)|_{\mathcal {S}})$ be the i’th smallest eigenvalues of *A* and $\tilde{\Phi }(A)|_{\mathcal {S}}$, respectively. Then for all $1\le i \le d^n$ and all *j* such that $(i-1)(p+q)+1\le j\le i(p+q)$,

149 $$\begin{aligned} |\lambda _j(\tilde{\Phi }(A)|_{\mathcal {S}}) - f(A)\lambda _i(A) | \le k(A)\epsilon . \end{aligned}$$

where the integers *p*, *q* and $f(\cdot )$ is the function appearing the corresponding exact duality map.

##### Proof

Let $\Phi$ be the exact duality map which is $\epsilon$-close to the restricted $\tilde{\Phi }$ and $\eta$-close to the local duality. For any *i*, *j* satisfying the above inequalities, $\lambda _j\left( \Phi (A)\right) = f(A)\lambda _i(A)$ from axiom (iii) of Definition 3 of exact dualities. Combining this with Weyl’s inequality ($|\lambda _j(A)-\lambda _j\left( B \right) |\le \Vert A- B\Vert$) gives,

150 $$\begin{aligned} |\lambda _j(\tilde{\Phi }(A)|_{\mathcal {S}})-f(A)\lambda _i(A) |&= |\lambda _j(\tilde{\Phi }(A)|_{\mathcal {S}})-\lambda _j\left( \Phi (A) \right) | \end{aligned}$$

151 $$\begin{aligned}&\le \Vert \tilde{\Phi }(A)|_{\mathcal {S}} - \Phi (A)\Vert \end{aligned}$$

152 $$\begin{aligned}&\le k(A)\epsilon . \end{aligned}$$

$$\square $$

#### Thermal Properties

Similarly Definition 10 includes a partition-function-like statement motivated by requiring dual thermal properties. Approximate duality mappings preserve partition functions of a given Hamiltonian up to a controllable error, when the restricted subspace is taken to be the low energy subspace of the Hamiltonian in question.

##### Proposition 28

(Approximate partition functions). Let the Hamiltonian *H* act on $\left( \mathbb {C}^d \right) ^{\otimes n}$ and $\tilde{\Phi }$ be the $(\mathcal {S},\epsilon , \eta )$-duality map into $(\mathbb {C}^{d'} )^{\otimes m}$, where $\mathcal {S}$ is the low energy subspace of *H* with energy less than $\Delta$. The relative error in the dual partition functions is given by,

153 $$\begin{aligned} \frac{|\mathcal {Z}_{\tilde{\Phi }(H)}(\beta ) - (p+q)\mathcal {Z}_{H}(f(H)\beta ) |}{(p+q)\mathcal {Z}_{H}(f(H)\beta )} \le \frac{(d')^m e^{-\beta \Delta }}{(p+q)d^n e^{-\beta f(H)\Vert H\Vert }} + \left( e^{\beta k(H)\epsilon }-1 \right) ,\nonumber \\ \end{aligned}$$

where the integers *p*, *q* and $f(\cdot )$ is the function in the corresponding exact duality map.

##### Proof

By axiom (iii) Definition 10 of an exact duality $(p+q)\textrm{tr} \left[ e^{-\beta f(H)H} \right] = \textrm{tr}\left[ e^{-\beta \Phi (H)}\right]$. Therefore,

154 $$\begin{aligned} \frac{|\mathcal {Z}_{\tilde{\Phi }(H)}(\beta ) - (p+q)\mathcal {Z}_{H}(f(H)\beta ) |}{(p+q)\mathcal {Z}_{H}(f(H)\beta )}&= \frac{|\textrm{tr}\left[ e^{-\beta \tilde{\Phi }(H)} \right] - (p+q)\textrm{tr}\left[ e^{-\beta f(H)H} \right] |}{(p+q)\textrm{tr}\left[ e^{-\beta f(H)H} \right] } \end{aligned}$$

155 $$\begin{aligned}&= \frac{|\textrm{tr}\left[ e^{-\beta \tilde{\Phi }(H)} \right] - \textrm{tr}\left[ e^{-\beta \Phi (H)} \right] |}{(p+q)\textrm{tr}\left[ e^{-\beta f(H)H} \right] } \end{aligned}$$

156 $$\begin{aligned}&\le \frac{ |\textrm{tr}\left[ e^{-\beta \tilde{\Phi }(H)} \right] - \textrm{tr}\left[ e^{-\beta \left. \tilde{\Phi }(H)\right| _{\mathcal {S}} }\right] |}{(p+q)\textrm{tr}\left[ e^{-\beta f(H)H} \right] }\nonumber \\&\quad + \frac{ |\textrm{tr}\left[ e^{-\beta \left. \tilde{\Phi }(H)\right| _{\mathcal {S}} }\right] - \textrm{tr}\left[ e^{-\beta \Phi (H)} \right] |}{\textrm{tr}\left[ e^{-\beta \Phi (H)} \right] } . \end{aligned}$$

Bounding the numerator and denominator of the first term:

157 $$\begin{aligned} |\textrm{tr}\left[ e^{-\beta \tilde{\Phi }(H)} \right] - \textrm{tr}\left[ e^{-\beta \left. \tilde{\Phi }(H)\right| _{\mathcal {S}} }\right] |&\le (d')^m e^{-\beta \Delta }. \end{aligned}$$

158 $$\begin{aligned} \textrm{tr}\left[ e^{-\beta f(H)H} \right]&\ge d^n e^{-\beta f(H)\Vert H\Vert }. \end{aligned}$$

The second term is bounded by considering eigenvalues. Let $\lambda _l$ be the *l*’th eigenvalue of $\left. H'\right| _{\mathcal {S}}$ in non-decreasing order. Then by the argument in Proposition 27, the *l*’th eigenvalue of $\Phi (H)$ (in the same order) is given by $\lambda _l + k(H)\epsilon _l$ where $|\epsilon _l |\le \epsilon$ for all *l*. Hence,

159 $$\begin{aligned} |\textrm{tr}\left[ e^{-\beta \left. \tilde{\Phi }(H)\right| _{\mathcal {S}} }\right] - \textrm{tr}\left[ e^{-\beta \Phi (H)} \right] |&\le \sum _l |e^{-\beta \lambda _l} - e^{-\beta (\lambda _l + k(H)\epsilon _l)} | \end{aligned}$$

160 $$\begin{aligned}&=\sum _l e^{\beta (\lambda _l+k(H)\epsilon _l)} |e^{\beta k(H)\epsilon _l}-1 | \end{aligned}$$

161 $$\begin{aligned}&\le (e^{\beta k(H)\epsilon }-1)\textrm{tr}\left[ e^{-\beta \Phi (H)} \right] . \end{aligned}$$

Combining the above with Eqs. (157) and (158) gives the result. $\square$

#### Time Dynamics

Definition 8 demanded consistent time dynamics for exact duality mappings as a constraint to specify the form of the corresponding state map. As expected when considering approximate duality maps this statement is relaxed, such that time dynamics of the two systems is close up to an error that increases with time.

##### Proposition 29

(Approximate time dynamics). Let $\tilde{\Phi }$ be a $(\mathcal {S},\epsilon ,\eta )$-approximately local duality map with corresponding exact duality $\Phi (\cdot ) = f(\cdot )\mathcal {E}(\cdot )$. Given a Hamiltonian *H* such that $\mathcal {S}$ is the low energy subspace with eigenvalues $< \Delta$. Then for any density matrix $\rho$ in the encoded subspace, such that $\Phi (\mathbb {I})\rho = \rho$, the time dynamics of the approximate duality mapping is close to that of the exact mapping:

162 $$\begin{aligned} \Vert e^{-i\tilde{\Phi }(H)t}\rho e^{i\tilde{\Phi }(H)t} - e^{-i\Phi (H)t}\rho e^{i\Phi (H)t}\Vert _1 \le 2\epsilon k(H) t + \eta . \end{aligned}$$

This follows from an identical argument as Proposition 29 from [11], applying instead $\Vert \left. \tilde{\Phi }(H)\right| _{\mathcal {S}} - \Phi (H)\Vert \le k(H)\epsilon$ at the final step.

## Appendix A: Proof of Lemma 14

### Lemma 14

(Entropy of mixtures of mixed states). Given a density operator, $\rho _\mathcal {A} = \sum _{x=1}^k p_x \rho _x$, that is a probabilistic mixture of mixed states $\rho _x$, with $p_x\in [0,1]$ and $\sum _x p_x =1$. The von Neumann entropy of $\rho _\mathcal {A}$ obeys the following equality,

$$\begin{aligned}S(\rho _\mathcal {A}) = \sum _x p_x S(\rho _x) -\sum _x p_x \log p_x,\end{aligned}$$

if and only if $\rho _x$ have orthogonal support. I.e. $\textrm{tr}[\rho _x \rho _y]=0$ for all $x\ne y$.

### Proof

Write each mixed state as a sum of pure states:

163 $$\begin{aligned} \rho _x = \sum _{j=1}^m \lambda _j^{(x)} \left| \phi _j^{(x)}\right\rangle \left\langle \phi _j^{(x)}\right| , \end{aligned}$$

where $\{ \left| \phi _j^{(x)}\right\rangle \}_{j=1}^m$ form an orthogonal basis for a given *x*, but in general ${\langle \phi _i^{(x)}|\phi _j^{(y)}\rangle }\ne 0$ for $x\ne y$. The full density operator with these expansions reads:

164 $$\begin{aligned} \rho _\mathcal {A} = \sum _{x=1}^k \sum _{j=1}^m p_x \lambda _j^{(x)} \left| \phi _j^{(x)}\right\rangle \left\langle \phi _{j}^{(x)}\right| . \end{aligned}$$

Introduce a Hilbert space, $\mathcal {H}_\mathcal {R}$, with $\dim (\mathcal {H}_\mathcal {R})= mk$ and an orthonormal basis labelled by $\left| xj\right\rangle _\mathcal {R}$. Consider a purification of $\rho _\mathcal {A}$,

165 $$\begin{aligned} \left| \mathcal{A}\mathcal{R}\right\rangle = \sum _{x,j}\sqrt{p_x \lambda _j^{(x)}}\left| \phi _j^{(x)}\right\rangle _\mathcal {A} \otimes \left| xj\right\rangle _\mathcal {R}, \end{aligned}$$

where

166 $$\begin{aligned} \rho _\mathcal {A}&= \textrm{tr}_\mathcal {R} \left[ \left| \mathcal{A}\mathcal{R}\right\rangle \left\langle \mathcal{A}\mathcal{R}\right| \right] = \sum _{x,j} p_x \lambda _j^{(x)} \left| \phi _j^{(x)}\right\rangle \left\langle \phi _j^{(x)}\right| , \end{aligned}$$

and

167 $$\begin{aligned} \rho _\mathcal {R}&= \textrm{tr}_\mathcal {A} \left[ \left| \mathcal{A}\mathcal{R}\right\rangle \left\langle \mathcal{A}\mathcal{R}\right| \right] \end{aligned}$$

168 $$\begin{aligned}&= \sum _{x,j,x',j'} \sqrt{p_x \lambda _j^{(x)}} \sqrt{p_{x'}\lambda _{j'}^{(x')}} {\langle \phi _j^{(x)}|\phi _{j'}^{(x')}\rangle }_\mathcal {A} \left| xj\right\rangle \left\langle x'j'\right| . \end{aligned}$$

Also define

169 $$\begin{aligned} \rho _\mathcal {R'}:= \sum _{x,j} p_x \lambda _j^{(x)} \left| xj\right\rangle \left\langle xj\right| . \end{aligned}$$

The relative entropy between the two reservoir states is given by

170 $$\begin{aligned} S(\rho _\mathcal {R}||\rho _\mathcal {R'})&:= \textrm{tr}\rho _\mathcal {R} \log \rho _\mathcal {R} - \textrm{tr}\rho _\mathcal {R}\log \rho _\mathcal {R'} \end{aligned}$$

171 $$\begin{aligned}&= - S(\rho _\mathcal {R}) - \textrm{tr}\rho _\mathcal {R}\log \rho _\mathcal {R'} \end{aligned}$$

172 $$\begin{aligned}&= - S(\rho _\mathcal {A}) - \textrm{tr}\rho _\mathcal {R}\log \rho _\mathcal {R'}. \end{aligned}$$

Where the last line uses $S(\rho _\mathcal {R}) = S(\rho _\mathcal {A})$. Since $\left| xj\right\rangle$ forms an orthogonal basis $\log \rho _\mathcal {R'} = \sum _{x,j}\log \left( p_x \lambda _j^{(x)} \right) \left| xj\right\rangle \left\langle xj\right|$. Further algebraic manipulation of the last term results in,

173 $$\begin{aligned} \textrm{tr}\left[ \rho _\mathcal {R} \log \rho _\mathcal {R'}\right]&= \sum _{j,x} \log \left( p_x \lambda _j^{(x)} \right) \textrm{tr}\left( \rho _\mathcal {R}\left| xj\right\rangle \left\langle xj\right| \right) \end{aligned}$$

174 $$\begin{aligned}&= \sum _{x,j} \log \left( p_x \lambda _j^{(x)} \right) {\langle xj| \rho _\mathcal {R}|xj\rangle } \end{aligned}$$

175 $$\begin{aligned}&= \sum _{x,j} p_x \lambda _j^{(x)} \log \left( p_x \lambda _j^{(x)} \right) \end{aligned}$$

176 $$\begin{aligned}&= \sum _{x,j} p_x \lambda _j^{(x)}\log p_x + \sum _{x,j} p_x \lambda _j^{(x)}\log (\lambda _j^{(x)}) \end{aligned}$$

177 $$\begin{aligned}&= \sum _x p_x \log p_x + \sum _x p_x \sum _j \lambda _j^{(x)} \log \lambda _j^{(x)} \end{aligned}$$

178 $$\begin{aligned}&= \sum _x p_x \log p_x - \sum _x p_x S(\rho _x). \end{aligned}$$

We arrive at an expression for the entropy of our mixture of mixed states,

179 $$\begin{aligned} S(\rho _\mathcal {A}) = \sum _x p_x S(\rho _x) - \sum _x p_x \log p_x - S(\rho _\mathcal {R}||\rho _\mathcal {R'}). \end{aligned}$$

Since the relative entropy $S(\rho _\mathcal {R}||\rho _\mathcal {R'})=0$ if and only if $\rho _\mathcal {R} = \rho _\mathcal {R'}$, the expressions for $\rho _\mathcal {R}$ and $\rho _\mathcal {R'}$ in Eqs. (168), Eq. (169), respectively, imply that the two density matrices are equal if and only if the corresponding vectors $\left| \phi _j^{(x)}\right\rangle$ form an orthogonal set (given *j*, *x* such that $\lambda _j^{(x)}\ne 0$). This is equivalent to stating that the mixed states $\rho _x$ must have orthogonal support. $\square$

## Appendix: B Proof of Lemma 15

### Lemma 15

(Pure states mapped to orthogonal density matrices). Let $\{ \sigma _i \}_{i=1}^d$ be a set of orthogonal pure states that forms a basis in $\mathcal {H}$, with $\sigma _i \in P_1(\mathcal {H})$. Let the map $\phi : \mathcal {S}(\mathcal {H}_n)\mapsto \mathcal {S}(\mathcal {H}_{\alpha n})$, be

- Entropy preserving up to an additive constant, $S(\phi (\rho )) = S(\rho ) + \log \alpha$;
- Convex, $\phi (t\rho + (1-t)\sigma ) = t \phi (\rho ) + (1-t) \phi (\sigma )$. Where $t\in [0,1]$ and $\rho ,\sigma \in \mathcal {S}(\mathcal {H})$.

The image of this set under the map is a new set, $\{\phi (\sigma _i) \}_{i=1}^d$, with orthogonal support.

### Proof

Any state in $\mathcal {S}(\mathcal {H})$ can be written as a linear combination of the set of pure states. The map $\phi$ obeys entropy relation (a) so,

180 $$\begin{aligned} S\left( \phi \left( \sum _{i=1}^d \lambda _i \sigma _i\right) \right) = S\left( \sum _{i=1}^d \lambda _i \sigma _i \right) + \log \alpha . \end{aligned}$$

Since $\{ \sigma _i\}_{i=1}^d$ have orthogonal support, Lemma 14 can be applied to the first term:

181 $$\begin{aligned} S\left( \phi (\sum _{i=1}^d \lambda _i \sigma _i) \right) = \sum _{i=1}^d \lambda _i S(\sigma _i) - \sum _{i=1}^d \lambda _i \log \lambda _i + \log \alpha . \end{aligned}$$

Reusing the entropy preserving property of $\phi$, this time with a sum over pure states with $S(\sigma _i)=0$, $S(\phi (\sigma _i))=\log \alpha$ for all *i*. Since $\sum _{i=1}^d \lambda _i = 1$, $\sum _{i=1}^d \lambda _i S(\phi (\sigma _i))=\log \alpha$, thus

182 $$\begin{aligned} S\left( \phi \left( \sum _{i=1}^d \lambda _i \sigma _i\right) \right) = - \sum _{i=1}^d \lambda _i \log \lambda _i + \sum _{i=1}^d \lambda _i S(\phi (\sigma _i)). \end{aligned}$$

Since there is an equality, the only if direction of Lemma 14 implies that $\{ \phi (\sigma _i)\}$ must have orthogonal support. $\square$

## Appendix C: Vanishing Off-Diagonal Matrix Elements

This appendix demonstrates that if a Hermitian projector, , has $D=\textbf{0}$ then necessarily $B=C=\textbf{0}$.

To show this, two properties of $\Pi$ are useful,

1. Projectors are idempotent, $\Pi ^2 = \Pi$:
  183 $$\begin{aligned} \left( \begin{array}{cc} A &{} B \\ C &{} 0 \end{array} \right) \left( \begin{array}{cc} A &{} B \\ C &{} 0 \end{array} \right) = \left( \begin{array}{cc} A^2 + BC &{} AB \\ CA &{} CB \end{array} \right) = \left( \begin{array}{cc} A &{} B \\ C &{} 0 \end{array} \right) , \end{aligned}$$
  therefore $$CB=\textbf{0}$$.
2. Hermitian operators are self-adjoint, $\Pi ^\dagger = \Pi$:
  184 $$\begin{aligned} \left( \begin{array}{cc} A^\dagger &{} C^\dagger \\ B^\dagger &{} 0 \end{array} \right) = \left( \begin{array}{cc} A &{} B \\ C &{} 0 \end{array} \right) , \end{aligned}$$
  therefore $$B^\dagger = C$$.

Putting these together gives $BB^\dagger =\textbf{0}$. The eigenvalues and eigenvectors of adjoint matrices are related by $Bv_i=\lambda _i v_i$ and $B^\dagger v_i = \overline{\lambda _i}v_i$. Therefore, since $|\lambda _i|=0$ for all *i* in the diagonal basis, $B=C=\textbf{0}$.

## Appendix D: Matching Up of Spectra

The proof of Lemma 7 claims that the trivial permutation $\sigma (k)=k$ is the only allowed case for

185 $$\begin{aligned} \frac{G(A)}{f(A)} h(\mu _{\sigma (j)}) g(c_{\sigma (j)}P_{\sigma (j)}) f(c_{\sigma (j)}P_{\sigma (j)})c_{\sigma (j)} = \mu _jc_j. \end{aligned}$$

Note that there are $\dim (A)$ such equations corresponding to selecting for the different spectral projectors.

One could conceive that the permutation depends on the operator as well as the map, so that for a different operator *B*,

186 $$\begin{aligned} \frac{G(B)}{f(B)} h\left( \mu ^{(B)}_{\nu (j)}\right) g \left( c^{(B)}_{\nu (j)}P^{(B)}_{\nu (j)}\right) f\left( c^{(B)}_{\nu (j)}P^{(B)}_{\nu (j)}\right) c^{(B)}_{\nu (j)} = \mu ^{(B)}_j c^{(B)}_j, \end{aligned}$$

where $\sigma (j)\ne \nu (j)$. However, the spectral projectors are analytic functions of the matrix [26] (assuming for now that the matrices are non-degenerate). Any two matrices *A* and *B* can be connected by a smooth path, ruling out a change in the permutation which would require a discontinuous jump. Therefore, the permutation must be consistent for all inputs to the map. The exception to this case is where there are degeneracies, but as we are only interested in equating eigenvalues, a permutation of degenerate eigenvalues within the degenerate spectral projectors has no affect.

To justify that the permutation must be trivial everywhere we show by contradiction that it must be trivial for any *A*. Consider the following analytic change to the operator $A\rightarrow A'$: one eigenvalue is changed $\mu _k'c_k'= \mu _kc_k+\delta$ ($\delta >0$), while all other eigenvalues $\mu _{j\ne k}'c_{j\ne k}'=\mu _{j\ne k}c_{j\ne k}$, and all spectral projectors $P'_{j} = P_j$ are held unchanged. For this new operator, a similar set of equations hold with the same permutation:

187 $$\begin{aligned} \frac{G(A')}{f(A')} h(\mu '_{\sigma (j)}) g( c'_{\sigma (j)}P_{\sigma (j)})f(c'_{\sigma (j)}P_{\sigma (j)})c'_{\sigma (j)} = \mu '_j c'_j. \end{aligned}$$

For all $j\ne 1$ such that $\sigma (j)\ne 1$

188 $$\begin{aligned} \frac{G(A)}{f(A)}\frac{f(A')}{G(A')} = \frac{h(\mu _{\sigma (j)})g( c_{\sigma (j)}P_{\sigma (j)})f(c_{\sigma (j)}P_{\sigma (j)})c_{\sigma (j)}}{h(\mu _{\sigma (j)}) g( c_{\sigma (j)}P_{\sigma (j)})f(c_{\sigma (j)}P_{\sigma (j)})c_{\sigma (j)}} =1. \end{aligned}$$

Assume for contradiction that $\sigma (k)= j \ne k$, so that there is some non-trivial permutation and dual spectral projectors are not paired with eigenvalues related by *f*(*A*). Then

189 $$\begin{aligned} \frac{G(A)}{f(A)}h(\mu _j) g(c_jP_j)f(c_jP_j)c_j&= \mu _kc_k \end{aligned}$$

190 $$\begin{aligned} \frac{G(A')}{f(A')}h(\mu _j) g(c_jP_j)f(c_jP_j)c_j&= \mu _kc_k + \delta . \end{aligned}$$

Therefore, $\frac{G(A')}{f(A')}\frac{f(A)}{G(A)}\mu _kc_k = \mu _kc_k + \delta$ and $\delta =0$, contradicting $\delta >0$ and the trivial permutation is the only allowed case.

## Appendix E: Kramers–Wannier Duality

The Kramer–Wannier duality links two 2d Ising models, one at low temperature (strong interaction strength) with another at high temperature (weak interaction strength). The duality is identified by computing the partition function of both systems in their respective limits. This appendix outlines the Kramer–Wannier duality and how it arises, based on [1, 27]. Final we show explicitly how it lies outside the original simulation framework of [11] and how can be placed in our more general framework of duality.

### E.1 Low-Temperature Expansion

The Ising model on an *N* site lattice is governed by the Hamiltonian $H = -J \sum _{\langle i, j \rangle }\sigma _i \sigma _j$. Consider the isotropic case where the interaction strength *J* is the same across both horizontal and vertical directions, $K:=\beta J$. If $K>0$ the model is ferromagnetic and the ground state will have all spins aligned. In the low-temperature regime, the system is dominated by its ground state. The expansion for the partition function at low temperature is given by the ground state configuration plus low energy fluctuations—i.e. 1, 2, 3,... spins aligned anti-parallel. The additional energy cost of one flipped spin in a 2d lattice is $4\times 2K$ and that of two flipped spins in a block is $6 \times 2K$. Counting the degeneracies of these states the partition function is given by,

191 $$\begin{aligned} Z \approx 2e^{\text {no. bonds total} \times K} \left[ 1 + Ne^{-4\times 2K} + 2N e^{-6 \times 2K} +... \right] . \end{aligned}$$

The energy cost comes from the domain wall boundary between the regions of anti-parallel spins. In the thermodynamic limit $N\rightarrow \infty$, the multiplicities become insignificant and the partition function can be written as

192 $$\begin{aligned} Z \approx 2e^{\text {no. bonds total} \times K} \sum _{\text {islands of} -\text {ve spins}} e^{-2K \times \text {perimeter of island}}. \end{aligned}$$

The terms in this summation can be represented graphically by creating islands of increasingly large regions of anti-aligned spin.

### E.2 High-Temperature Expansion

The high-temperature expansion starts instead with independent spins and the partition function is expanded in powers of $\beta$. A convenient simplification is to expand in powers of $\tanh K$ instead. This is equivalent to doing a high-temperature expansion since $\tanh K$ is less than 1 (except when $\beta \rightarrow \infty$) so in the high-temperature region powers of $\tanh K$ are increasingly small. Since $(\sigma _i\sigma _j)^2=1$ the bond $\langle i,j\rangle$ Boltzmann factor can be rewritten as

193 $$\begin{aligned} e^{K\sigma _i\sigma _j}&= \frac{e^{K}+e^{-K}}{2} + \frac{(e^{K}-e^{-K})}{2} \sigma _i \sigma _j \end{aligned}$$

194 $$\begin{aligned}&= \cosh K (1+\tanh K \sigma _i \sigma _j). \end{aligned}$$

Applying this transformation to the partition function gives

195 $$\begin{aligned} Z&= \sum _{\{\sigma _i \}} e^{K\sum _{\langle i, j \rangle }\sigma _i \sigma _j} \end{aligned}$$

196 $$\begin{aligned}&= (\cosh K)^\text {no. bonds} \sum _{\{ \sigma _i\}} \prod _{\langle i, j \rangle } (1+\tanh K \sigma _i \sigma _j). \end{aligned}$$

The product $\prod _{\langle i,j \rangle } (1+\tanh K \sigma _i \sigma _j) = (1+\tanh K \sigma _a \sigma _b)(1+\tanh K \sigma _a \sigma _c)(1+\tanh K \sigma _a \sigma _d)...$ generates $2^{N_b}$ terms where $N_b$ is the number of bonds in the lattice. Each term in the sum can again be represented as a graph: for all edges in the lattice draw a line on the edge (*i*, *j*) if there is a factor of $\tanh K \sigma _i \sigma _j$ (an occupied bond) and draw no line if the term in the expansion is 1 (an unoccupied bond). Each term in the expansion of the product is of the form

197 $$\begin{aligned} (\tanh K )^\text {no. occupied bonds}\sigma _1^{p_1} \sigma _2^{p_2} \sigma _3^{p_3}... \sigma _N^{p_N}. \end{aligned}$$

Now we perform the sum over each spin being $\pm 1$. Summing over $\sigma _i$ gives a factor of 2 if $p_i$ even and 0 if $p_i$ odd. Therefore, only graphs where every site has an even number of occupied legs is non-vanishing. These graphs form closed paths on the lattice. The high-temperature series expansion is then given by

198 $$\begin{aligned} Z = 2^N \times (\cosh K)^\text {no. bonds total} \sum _\text {closed graphs} (\tanh K)^\text {no. occupied bonds in the graph}.\nonumber \\ \end{aligned}$$

### E.3 Free Energy Duality

Taking the thermodynamic limit, so $N\rightarrow \infty$, the total number of bonds in the lattice becomes $\approx 2N$. Make the couplings strengths for the models in the different temperature regimes distinct: *K* in the high-temperature expansion, $\tilde{K}$ in the low-temperature expansion. The duality is identified by comparing the low- and high-temperature series expansions:

199 $$\begin{aligned} \text {Low temp:} \quad&Z(\tilde{K}) = 2e^{2N\tilde{K}}\sum _\text {islands of -ve spin} e^{-2\tilde{K} \times \text {perimeter of islands}} \end{aligned}$$

200 $$\begin{aligned} \text {High temp:} \quad&Z(K) = 2^N (\cosh K)^{2N} \sum _{\text {closed graphs}} \tanh K^{\text {length of graph}}. \end{aligned}$$

There is a correspondence between the two sums since islands of sites can be considered as closed graphs and vice versa. They differ only at the boundaries, but in the thermodynamic limit this difference becomes negligable. Defining the function,

201 $$\begin{aligned} g(x):= \lim _{N\rightarrow \infty } \ln \sum _\text {closed graphs} x^\text {no. lines in the graph}, \end{aligned}$$

the arguments of *g* in each of the above sum are related by the duality condition

202 $$\begin{aligned} e^{-2\tilde{K}} \leftrightarrow \tanh K \qquad \tilde{K} = D(K) = -\frac{1}{2} \ln \tanh K. \end{aligned}$$

With the above function *g*, the free energies per particle can be written as:

203 $$\begin{aligned} \text {Low temp:} \quad&-\beta f_H = \frac{\ln Z(K)}{N} = 2K + \ln \left\{ e^{-4\times 2K} + 2e^{-4\times 6K} - \frac{5}{2} e^{-8\times 2K}+...\right\} \end{aligned}$$

204 $$\begin{aligned} \text {High temp:} \quad&-\tilde{\beta } f_{\tilde{H}} = \frac{\ln Z(\tilde{K})}{N} = \ln 2 + 2 \ln \cosh K + \ln \left\{ (\tanh K)^4+ ...\right\} . \end{aligned}$$

The duality condition, Eq. (202), then relates the two free energies by:

205 $$\begin{aligned} \tilde{\beta } f_{\tilde{H}} = \beta f_H + 2 \beta J - \ln \left[ 2 \cosh ^2\left( \tilde{\beta }\tilde{J}\right) \right] . \end{aligned}$$

Some algebra manipulates the free energy relation into a simpler form:

206 $$\begin{aligned} 2\beta J - \ln \left[ 2 \cosh ^2\left( \tilde{\beta }\tilde{J}\right) \right]&= \ln \left[ \frac{e^{2\beta J}}{2\cosh ^2\left( \ln \tanh ^{-1/2}J\beta \right) } \right] \end{aligned}$$

207 $$\begin{aligned}&= \ln \left[ \frac{e^{2\beta J}}{2\times (1/4) \times \left( \tanh ^{-1/2}J\beta + \tanh ^{1/2}J\beta \right) ^2 } \right] \end{aligned}$$

208 $$\begin{aligned}&= \ln \left[ \frac{e^{2\beta J}}{2\times (1/4) \times \left( \tanh ^{-1} J\beta + \tanh J\beta + 2\right) } \right] \end{aligned}$$

209 $$\begin{aligned}&= \ln \left[ \frac{e^{2\beta J}}{(1/2) \times \left( \frac{\cosh J\beta }{\sinh J\beta } + \frac{\sinh J\beta }{\cosh J\beta } + 2\right) } \right] \end{aligned}$$

210 $$\begin{aligned}&= \ln \left[ \frac{e^{2\beta J}}{ \left( \frac{\cosh ^2 J\beta + \sinh ^2 J\beta + 2sinh J\beta \cosh J \beta }{2 \sinh J\beta \cosh J \beta }\right) } \right] \end{aligned}$$

211 $$\begin{aligned}&= \ln \left[ \frac{e^{2\beta J}}{\left( \frac{\cosh 2J\beta + \sinh 2J\beta }{\sinh 2J\beta }\right) } \right] \end{aligned}$$

212 $$\begin{aligned}&= \ln \left[ \frac{e^{2\beta J}}{\left( \frac{e^{2J\beta } }{\sinh 2J\beta }\right) } \right] \end{aligned}$$

213 $$\begin{aligned}&= \ln \left[ \sinh 2J\beta \right] \end{aligned}$$

From the free energy, the partition functions can be related using $-\beta f = \ln Z$:

214 $$\begin{aligned} Z_{\tilde{H}}(\tilde{\beta })&= \exp \left[ -N\tilde{\beta }f_{\tilde{H}} \right] \end{aligned}$$

215 $$\begin{aligned}&= \exp \left[ -N\beta f_H - N \ln \sinh (2\beta J) \right] \end{aligned}$$

216 $$\begin{aligned}&= e^{\ln \sinh (2\beta J)^{-N}}Z_H(\beta ) \end{aligned}$$

217 $$\begin{aligned}&= \left[ \sinh (2\beta J)\right] ^{-N} Z_H(\beta ). \end{aligned}$$

### E.4 In This Framework

We consider the map on operators that relates the two dual Hamiltonians. The encoding part of the duality is simple as the Hilbert space is the same size so there are no copies ($p=1$) and the form of the operators is the same so the unitary is simply the identity. The more interesting part of the duality appears in the scale factor, which should be a function of the initial Hamiltonian only. In the partition function and time evolution operator, temperature and the Hamiltonian always appear as a product ($\beta H$). Since in the Ising model the Hamiltonian is proportional to the coupling constant *J*, for Ising type Hamiltonians there is a trivial duality condition $J\beta = J'\beta '$. We therefore have additional freedom in how we chose to construct the set of maps that correspond to different physical scenarios if one were to engineer this duality. The first choice is consistent with how the duality framework in this paper has been set out; however, the different approaches are mathematically equivalent.

In the first instance, we will view Kramer–Wannier through the lens of a strong–weak duality: equating the temperatures of the dual systems $\beta = \tilde{\beta }$. A strongly interacting Ising model with interaction strength *J* is dual to a weakly interacting Ising model with interaction strength $\tilde{J}\ne J$ at the same temperature. This leads to a non-trivial scaling function for the map on operators that depends both on the operator and the temperature of the system:

218 $$\begin{aligned} \Phi _H(H)&= -\frac{1}{2J\beta }\ln \tanh (J\beta ) \times \mathbb {I}(H) \end{aligned}$$

219 $$\begin{aligned}&= f(H, \beta ) \times \mathcal {E}(H), \end{aligned}$$

where $\mathcal {E}(H)=\mathbb {I}(H)$ is an encoding satisfying the axioms 1-3 from Theorem 1 and $f(H, \beta ) = -\frac{1}{2J\beta }\ln \tanh (J\beta )$. The coupling strength, *J*, can be written as a function of the Hamiltonian norm and *n* the number of lattice sites: $J= \frac{\Vert H\Vert }{2n(n-1)}$.

Another approach could be to fix the interaction strength $J = \tilde{J}$ and consider a high–low-temperature duality where physical properties of the two dual systems are evaluated at different temperatures $\beta \ne \tilde{\beta }$. This does not allow the duality to be manipulated into our framework since we do not allow a temperature map. Here, the map on operators is independent of temperature with a trivial scaling function, $f(H)=1$:

220 $$\begin{aligned} \Phi (H) = \mathbb {I}(H). \end{aligned}$$

This viewpoint introduces the necessity of a temperature map, $\Phi _\beta$, should map positive reals to positive reals and be compatible with the Hamiltonian map such that the duality condition is satisfied. We will allow the temperature map to additionally depend on Hamiltonian parameters so there is a consistent set of maps for one system. In order to satisfy Eq. (5), the temperature map is

221 $$\begin{aligned} \Phi _\beta (\beta )=-\frac{1}{2J}\ln \tanh (J\beta ). \end{aligned}$$

This is perhaps the more immediate viewpoint from the Kramer–Wannier literature, but both approaches are mathematically equivalent. In fact using any interpolation of these two cases is also valid. We could consider two Ising models with different interaction strengths at different temperatures, as long as the product obeys Eq. (5). Furthermore, neither case fit into the original simulation framework in [11] for differing reasons. The first has a non-trivial scaling function, so that the spectra of two dual operators are not equal. The second has a non-trivial temperature map, so that the systems are only dual if considered at the appropriate temperature.

We can complete the description by providing a compatible map on states. Again we have choices. We could require the Born rule with respect to energy measurements should be preserved, or we could alternatively demand that thermal states map to thermal states. Starting with energy measurement outcomes, the expected behaviour

222 $$\begin{aligned} \textrm{tr}\left[ H \rho \right] = \frac{1}{f(H)} \textrm{tr}\left[ \Phi _H(H)\Phi _\text {state}(\rho ) \right] \end{aligned}$$

is achieved by a trivial mapping on states $\Phi _\text {states}(\rho )=\rho$. If instead we propose preserving Gibbs states,

223 $$\begin{aligned} \Phi _\text {state}\left( \frac{e^{-\beta H}}{Z_H(\beta )}\right) = \frac{e^{-\Phi _\beta (\beta )\Phi _H(H)} }{Z_{\Phi _H(H)}(\Phi _\beta (\beta ))}, \end{aligned}$$

then another choice for a map on states is $\Phi _\text {state}(\rho ) = \frac{\epsilon (\rho )}{\textrm{tr}(\epsilon (\rho ))}$ with $\epsilon (\rho ) = \rho ^{\frac{1}{2J\beta }\ln \tanh (J\beta )}$. These state mappings will preserve measurement outcomes and thermal states, respectively paired with either the strong–weak or high–low formulations described earlier.

## Footnotes

1 This theorem quoted here is informal and some technicalities have been omitted that can be found in the reference.

2 Note that Axiom (ii) is a slight abuse of notation since the map $\Phi$ is really a function of the ensemble $\{p_i,a_i \}$. However, the outcome should not depend on how you chose to construct the ensemble average. It will turn out later (see Lemma 7 for details) that consistency with the final axiom imposes additional constrains the allowed probability and observable rescaling functions, such that $\Phi$ is truly only a function of the ensemble average. But this is a non-trivial consequence of the *iteraction* between convexity and preservation of other physical properties; it is not required just by the duality of observable ensembles.

3 Since Eq. (84) is an analytic function, see, e.g. [23] p133.

4 An antiunitary operator is a bijective antilinear map $W:\mathcal {H}\mapsto \mathcal {H}$ of a complex Hilbert space such that $\langle Wx, Wy \rangle = \overline{\langle x, y \rangle }$ for all $x,y\in \mathcal {H}$ where the overline denotes complex conjugation.
